# LRRK2 interacts with the vacuolar-type H^+^-ATPase pump a1 subunit to regulate lysosomal function

**DOI:** 10.1093/hmg/ddz088

**Published:** 2019-04-30

**Authors:** Rebecca Wallings, Natalie Connor-Robson, Richard Wade-Martins

**Affiliations:** Oxford Parkinson’s Disease Centre, Department of Physiology, Anatomy and Genetics, University of Oxford, South Parks Road, Oxford OX1 3QX, UK

## Abstract

Lysosomal dysfunction lies at the centre of the cellular mechanisms underlying Parkinson’s disease although the precise underlying mechanisms remain unknown. We investigated the role of leucine-rich repeat kinase 2 (LRRK2) on lysosome biology and the autophagy pathway in primary neurons expressing the human *LRRK2-G2019S* or *LRKK2-R1441C* mutant or the human wild-type (*hWT-LRRK2*) genomic locus. The expression of LRRK2-G2019S or hWT-LRRK2 inhibited autophagosome production, whereas LRRK2-R1441C induced a decrease in autophagosome/lysosome fusion and increased lysosomal pH. *In vivo* data from the cortex and substantia nigra pars compacta of aged LRRK2 transgenic animals revealed alterations in autophagosome puncta number reflecting those phenotypes seen *in vitro.* Using the two selective and potent LRRK2 kinase inhibitors, MLi-2 and PF-06447475, we demonstrated that the LRRK2-R1441C-mediated decrease in autolysosome maturation is not dependent on LRRK2 kinase activity. We showed that hWT-LRRK2 and LRRK2-G2019S bind to the a1 subunit of vATPase, which is abolished by the LRRK2-R1441C mutation, leading to a decrease in a1 protein and cellular mislocalization. Modulation of lysosomal zinc increased vATPase a1 protein levels and rescued the LRRK2-R1441C-mediated cellular phenotypes. Our work defines a novel interaction between the LRRK2 protein and the vATPase a1 subunit and demonstrates a mode of action by which drugs may rescue lysosomal dysfunction. These results demonstrate the importance of LRRK2 in lysosomal biology, as well as the critical role of the lysosome in PD.

## Introduction

Parkinson’s disease (PD) is a common progressive neurodegenerative disease, affecting ~1–2% of the population over the age of 65 years(1). Mutations in the gene encoding leucine-rich repeat kinase 2 (*LRRK2*) are the most frequent cause of familial PD (2), and cases have an indistinguishable clinico-pathological phenotype from sporadic PD. *LRRK2* polymorphisms are also associated with increased risk of sporadic PD (3), suggesting that LRRK2 may provide understanding of the molecular mechanisms of both sporadic and genetic diseases. Pathogenic mutations are found in the enzymatic domains of the LRRK2 protein. The *LRRK2-G2019S* mutation in the kinase domain leads to an increase in kinase activity (4), a potential pathogenic mechanism of LRRK2 that is further supported by the protective effects of LRRK2 kinase inactivation (5,6). Located in the GTPase domain of LRRK2, Arg^1441^ is the second most common site of pathogenic LRRK2 substitutions (*LRRK2-R1441C/G*), leading to decreased GTP hydrolysis (7,8) and motor deficits and defects in dopaminergic neurotransmission in rodent models (9,10,11,12).

Lysosomes are cytoplasmic membrane-enclosed organelles containing hydrolytic enzymes that degrade macromolecules and cell components. Many factors regulate lysosomal function and subsequent protein degradation such as lysosomal pH (13), calcium release (14) and membrane trafficking (15). Lysosomal function decreases with age, with a rise in lysosomal pH (16). Lysosomal dysfunction has been shown to lead to the accumulation of α-synuclein (17), which may play a role in the build-up of Lewy bodies, the pathological hallmark of PD. Neurons are especially vulnerable to deficiencies in autophagy substrate clearance. Without the aid of cell division, which decreases the burden of intracellular waste in mitotic cells, neurons are largely dependent on autophagy to prevent the accumulation of cellular protein and damaged organelles. The crucial role of lysosomes in PD is reinforced by the observation that genes involved in lysosomal function, such as *VPS35* (18), *ATP6V0A1* (19), *ATP13A2* (20), *GBA* (21) and *LIMP2* (22), have repeatedly been associated with the disease.

Autophagy is a multifaceted pathway, from substrate recognition and autophagosome formation to autophagosome-lysosome fusion and lysosomal protein degradation. LRRK2 localizes to autophagic compartments and has been implicated in mechanisms underlying the regulations of these steps (23,24). Increased autophagic flux with LRRK2 kinase inhibition has been reported (25), suggesting an inhibitory effect of LRRK2 on autophagy induction. Local calcium release from lysosomes has been shown to be required for lysosome fusion (14). Alterations in the autophagy pathway caused by LRRK2 have been reported to be rescued by an NAADP receptor antagonist (26), suggesting a mechanistic role of LRRK2 in lysosomal calcium homeostasis. Lysosomal degradation is highly dependent on lysosomal pH and substantial shifts in pH can severely disrupt both degradative enzyme activity and fusion of autophagosomes and lysosomes (25). LRRK2 has been implicated in lysosomal pH regulation in different model systems (26,27). It is likely, therefore, that LRRK2 does not simply regulate autophagy induction as previously assumed, but is instead involved throughout the pathway, with a critical role at the lysosome.

To advance our understanding of the impact of *LRRK2* mutations on the autophagy/lysosomal pathway, primary neuronal cultures were prepared from *LRRK2* bacterial artificial chromosome (BAC) transgenic rats expressing either the human *LRRK2-G2019S*, or *LRRK2-R1441C*, or the wild-type (WT) form (*hWT-LRRK2*) of the entire human *LRRK2* genomic locus (9). Neurons expressing human LRRK2-G2019S or hWT-LRRK2 exhibit a decrease in the rate of autophagosome formation, whereas neurons expressing human LRRK2-R1441C, but not LRRK2-G2019S and hWT-LRRK2, demonstrate a deficit in autophagosome-lysosome fusion, leading to decreased lysosome-mediated degradation. These phenotypes were also observed *in vivo* in aged animals, with an increase in LC3 levels in both cortical neurons and dopamine neurons of the substantia nigra pars compacta of *LRRK2-R1441C* animals. *In vitro*, we found that the decrease in autolysosome maturation in *LRRK2-R1441C* neurons was not dependent on LRRK2 kinase activity, whereas the phenotypes observed in *LRRK2-G2019S* and *hWT-LRRK2* cortical neurons were kinase-dependent. LRRK2*-R1441C* cultures also exhibited decreased lysosomal acidity and alterations in lysosomal calcium dynamics. We found that hWT-LRRK2 interacts with the a1 subunit of the v-type H^+^ ATPase proton pump (vATPase a1), which is responsible for regulating lysosomal pH. Furthermore, the *LRRK2-R1441C* mutation abolished this interaction, dysregulating vATPase a1 protein expression and cellular localization. Finally, clioquinol, a zinc/copper ionophore, was capable of rescuing *LRRK2-R1441C* deficits through modulation of lysosomal zinc content. The increase in zinc content in lysosomes increased v-ATPase a1 subunit protein levels, corrected lysosomal pH, increased localized lysosomal calcium release and rescued the autophagosome/lysosome fusion deficit in *LRRK2-R1441C* neurons. These findings provide key insights both into the mechanisms by which LRRK2 impacts autophagy in PD and the modes of action of potentially-therapeutic drugs.

## Results

### *LRRK2-R1441C* neurons exhibit alterations in basal autophagy

**Figure 1:**
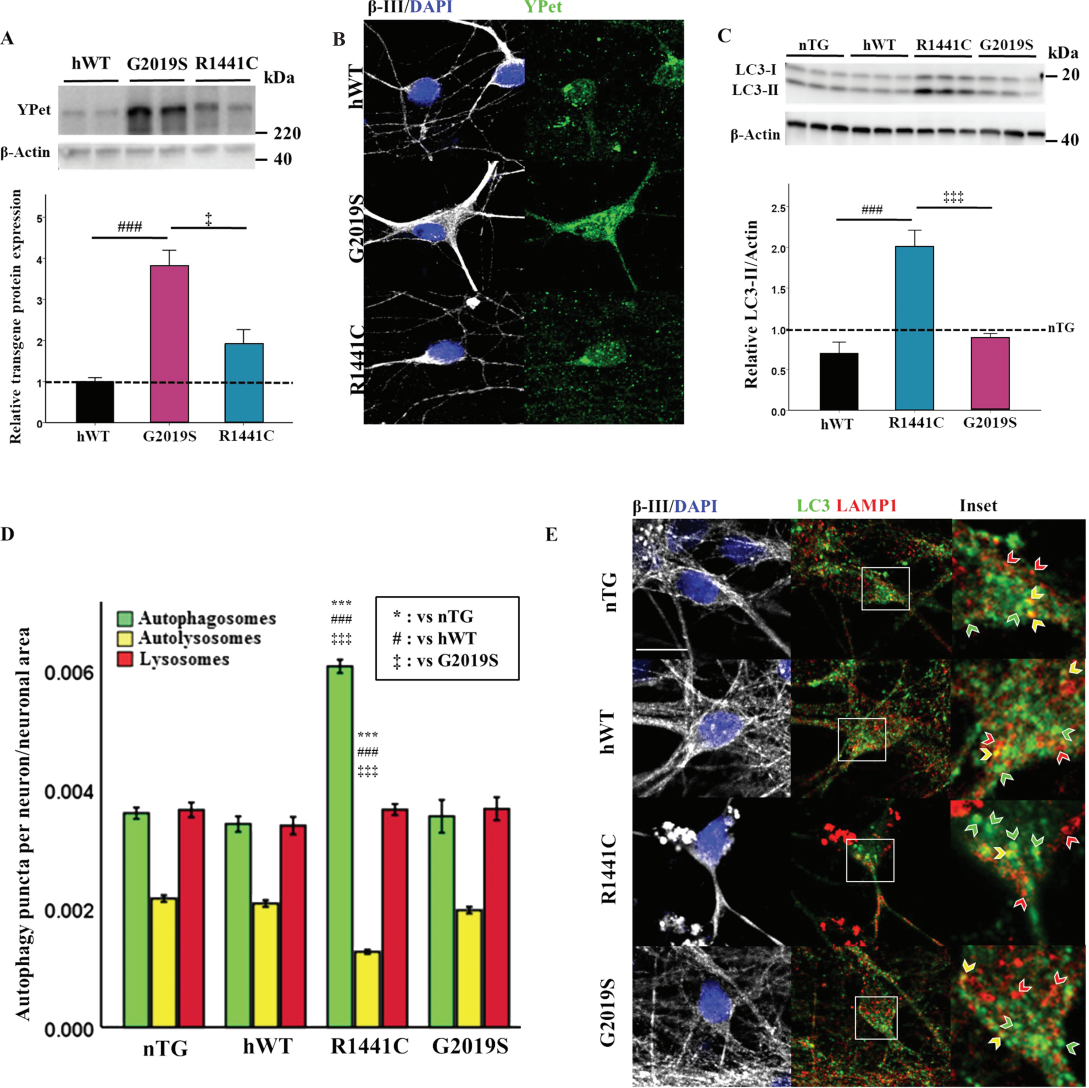
*LRRK2-R1441C* neurons exhibit alterations in basal autophagy. (**A**) Primary cortical neuronal cultures from P1 pups were generated and characterized. Western blots for YPet expression were quantified to assess transgene protein level and normalized to hWT levels. (**B**) Cultures at DIV 14 were stained for MAP2 and YPet to assess transgene expression in neurons**.** (**C**) Westerns blots for LC3-II were quantified and normalized to nTG littermate controls (dotted line = nTG normalized average; bars = mean +/− SEM; N = 3; ^*^P < 0.05, ^***^P < 0.001; one-way ANOVA, Tukey HSD post hoc). (**D, E**) DIV 14 primary cortical cultures were fixed and stained for β-III, LC3 and LAMP1, confocal images acquired and autophagy puncta number per neuron quantified and normalized to neuronal area. Bars represent mean +/− SEM. (N = 3; ^***^P < 0.001; Kruskal–Wallis non-parametric ANOVA, Bonferroni post hoc). ^*^, versus nTG; #, versus hWT; ‡ versus G2019S. Scale bar = 5 μm. Arrow heads: red, lysosome; yellow, autolysosome; green, autophagosome.

Primary cortical neuronal cultures from *LRRK2* BAC transgenic P1 rat pups were generated and characterized for expression of the human *LRRK2* transgene (using the transgenic N-terminal YPet protein tag) (9) and endogenous Lrrk2 protein expression. *hWT-LRRK2* and *LRRK2-R1441C* neurons express similar levels of each LRRK2 transgene, whereas *LRRK2-G2019S* cultures express a 2–3-fold higher level of transgene relative to both *hWT-LRRK2* and *LRRK2-R1441C* (Fig. 1A). Co-immunofluorescence staining for transgene protein and the neuronal marker MAP2 confirmed transgene presence in neurons (Fig.1B). *hWT-LRRK2* and *LRRK2-R1441C* transgene expression is 2–3-fold higher than endogenous Lrrk2 levels, and *LRRK2-G2019S* transgene expression is 5–6-fold higher (Supplementary Material, Fig. S1a), confirming previous published data from these animals (9).

**Figure 2:**
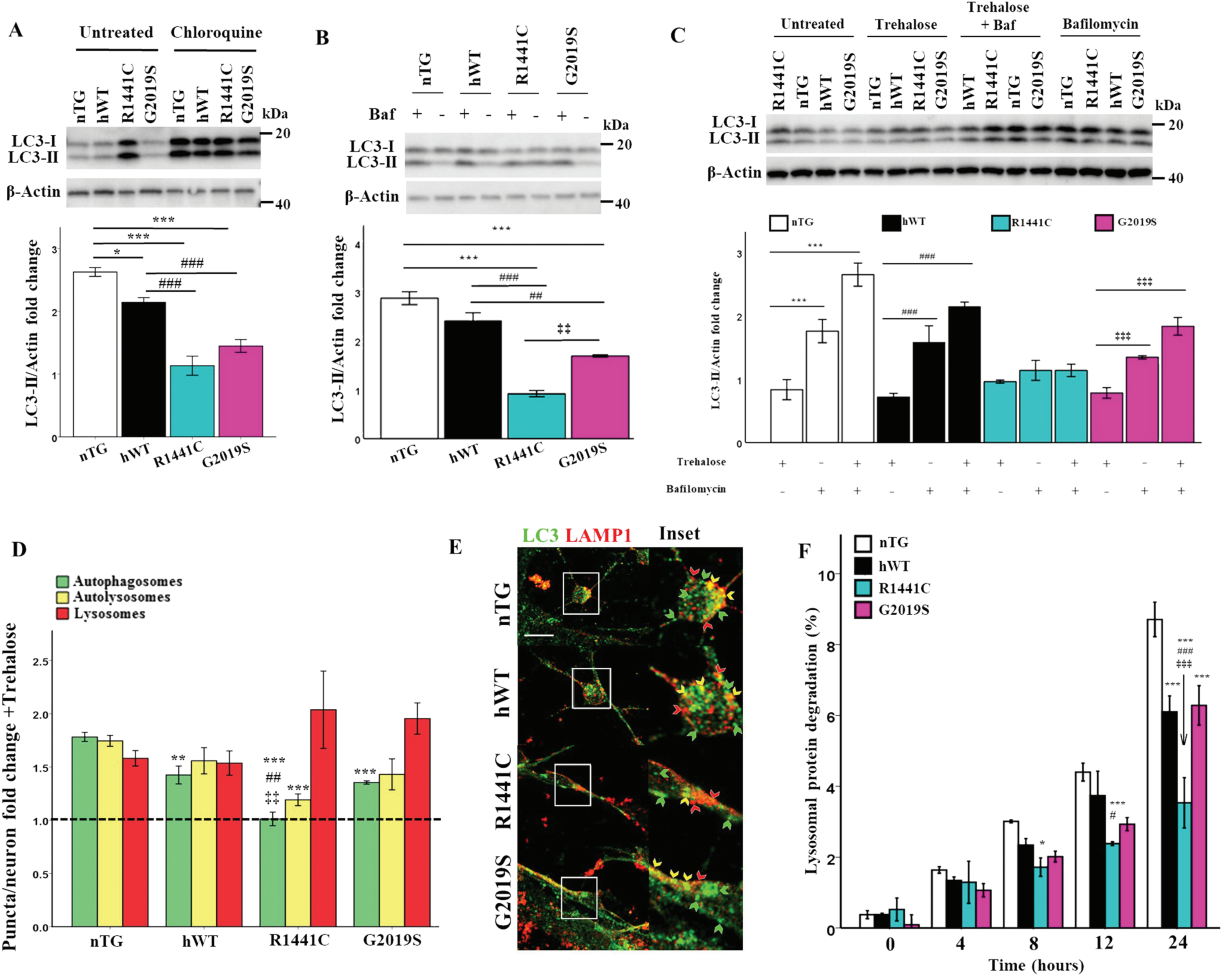
*LRRK2-R1441C* neurons have decreased autolysosome maturation, which cannot be rescued by lysosome biogenesis. (**A, B**) Western blots for LC3-II were quantified to assess LC3-II fold change from basal in response to treatment with 20 μm chloroquine for 8 h or 10 nm bafilomycin a1 for 2 h. (**C**) DIV 14 primary cortical neuronal cultures were treated with 500 μm trehalose for 24 h and/or 10 nm bafilomycin a1 for 2 h and western blots for LC3-II were quantified to measure changes in autophagic flux. Bars, mean +/− SEM (N = 3; ^*^P < 0.05; ^**^P < 0.005; ^***^P < 0.001; two-way ANOVA, Bonferroni post hoc). (**D, E**) DIV 14 primary cortical neuronal cultures were treated with 500 μm trehalose for 24 h, fixed and stained for LC3 and LAMP1 and puncta per neuron normalized to neuronal size quantified to measure fold change. Dotted line represents basal puncta number/neuron. Bars, mean +/− SEM (N = 3; ^**^P < 0.005; ^***^P < 0.001; Kruskal–Wallis non-parametric ANOVA, Bonferroni post hoc). Scale bar = 5 μm. Arrow heads: red, lysosome; yellow, autolysosome; green, autophagosome. (**F**) DIV 14 primary cortical neuronal cultures were incubated with radiolabelled valine for 48 h and CPM was measured over 24 h in untreated cultures or in the presence of chloroquine to calculate lysosomal protein degradation. Bars, mean +/− SEM (N = 3; ^*^P < 0.05; ^**^P < 0.005; ^***^P < 0.001; two-way ANOVA, Bonferroni post hoc). *, versus nTG; #, versus hWT; ‡, versus G2019S.

**Figure 3:**
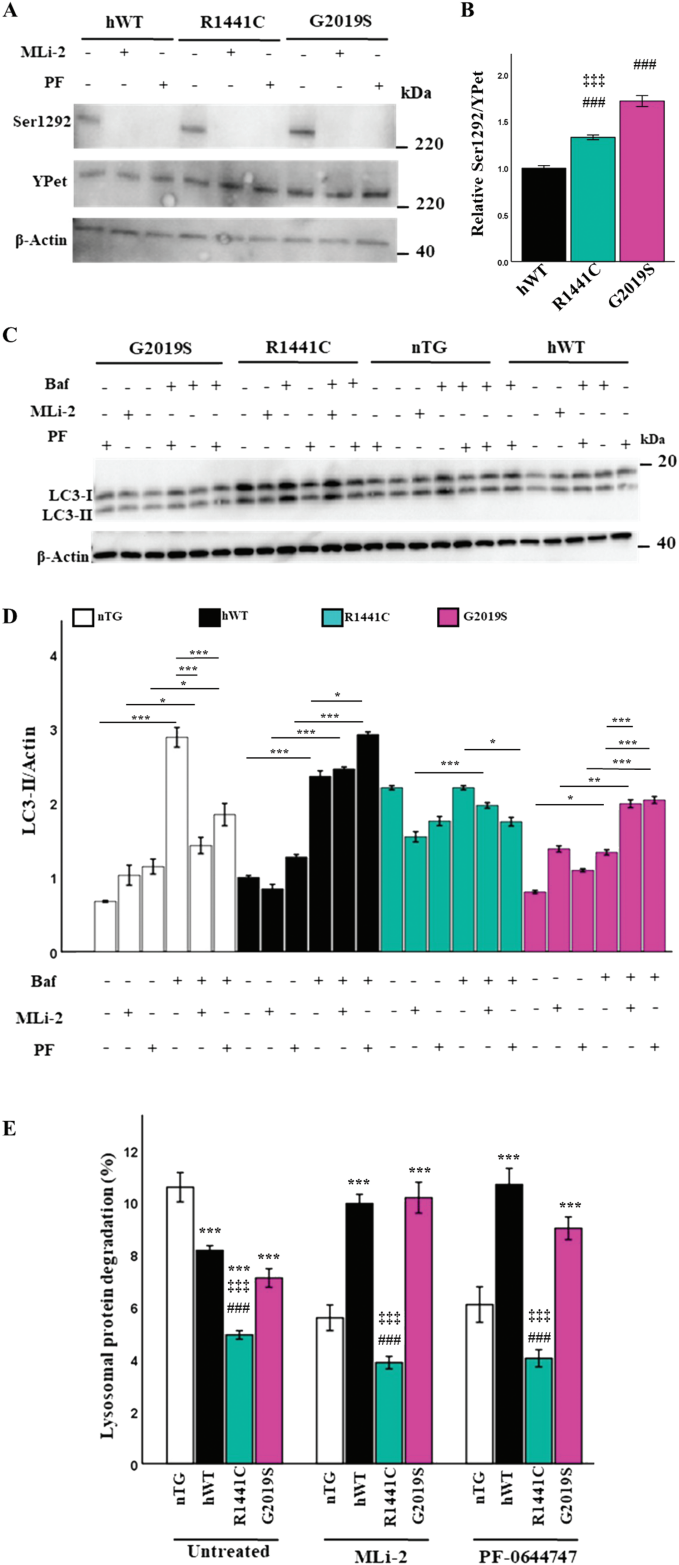
*LRRK2-R1441C* autophagy phenotype is not dependent on LRRK2 kinase activity (**A, B**) Primary cortical neuronal cultures at DIV 14 were treated with the LRRK2 kinase inhibitors (50 nm MLi-2 for 1 h or 50 nm PF-06447475 for 1 h) and assessed for expression of total YPet-tagged LRRK2 and phosphorylated LRRK2 at Ser1292 by western blot. (**B**) Proportion of Ser1292 phosphorylated YPet-tagged LRRK2 at basal levels was quantified by western blot. Bars, mean +/− SEM (N = 3; ^***^P < 0.001; Kruskal–Wallis non-parametric ANOVA, Bonferroni post hoc). ^*^, versus nTG; #, versus hWT; ‡, versus G2019S. (**C, D**) DIV 14 primary neuronal cultures were treated with 50 nm MLi-2, 50 nm PF-06447475 for 1 h or 10 nm Bafilomycin a1 for 2 h or in combination and LC3-II expression quantified to assess changes in autophagic flux. Bars, mean +/− SEM (N = 3; ^*^P < 0.05; ^**^P < 0.005; ^***^P < 0.001; two-way ANOVA, Bonferroni post hoc). (E) DIV 14 primary cortical neuronal cultures were incubated with radiolabelled valine for 48 h and CPM was measured over 24 h in untreated cultures or in the presence of MLi-2 or PF-064474745 to calculate lysosomal protein degradation. Bars, mean +/− SEM (N = 3; ^***^P < 0.001; three-way ANOVA, Bonferroni post hoc). ^*^, versus nTG; #, versus hWT; ‡, versus G2019S.

Expression of autophagy-related proteins was measured to assess basal autophagy levels in cortical neuronal cultures. *LRRK2-R1441C* neuronal cultures exhibited a significant increase in LC3-II (Fig.1C), although no significant differences in LAMP1 or p62 expression were observed (Supplementary Material, Fig. S1b,c). Primary cortical cultures contained a mixed population of cells, comprising 30–40% neurons (Supplementary Material, Fig. S1d,e). Co-immunofluorescence staining for the autophagy proteins LC3 and LAMP1 to quantify autophagosomes and autolysosomes demonstrated *LRRK2-R1441C* neurons showed a significant increase in autophagosome number and a significant decrease in autolysosome number (Fig. 1D and E; Supplementary Material, Fig. S1f). No significant changes in lysosome size were observed between genotypes (Supplementary Material, Fig. S1g).

### Assessing LC3 expression changes in cortical and midbrain tissue of aged animals

To understand whether the *LRRK2-R1441C*-dependent autophagy phenotype was present *in vivo* in brain tissue from aged animals, 22-month-old rat cortical sections were stained for LC3 and puncta quantified in MAP2+ cells (Supplementary Material, Fig. S2a). Cortical neurons from aged *LRRK2-R1441C* rats showed a significant increase in LC3 puncta number (Supplementary Material, Fig. S2b), with no change in puncta size (Supplementary Material, Fig. S2c). Midbrain sections containing the substantia nigra pars compacta were stained for LC3 and puncta quantified in tyrosine hydroxylase-positive (TH+) dopamine neurons (Supplementary Material, Fig. S2d). Interestingly, nigral dopamine neurons in the midbrain of both *LRRK2-R1441C* and *LRRK2-G2019S* animals demonstrated a significant increase in LC3 puncta (Supplementary Material, Fig. S2e), with no difference in LC3 puncta size (Supplementary Material, Fig. S2f). Therefore, although the effects of the *LRRK2-R1441C* mutation are present in different neuronal cell types, the effects of *LRRK2-G2019S* on the autophagy pathway might be cell-type dependent.

### *LRRK2-R1441C* neurons exhibit decreased autolysosome maturation

Steady state LC3-II protein levels and autophagy puncta counts can be difficult to interpret in terms of overall flux through the autophagy pathway as both induction of autophagy, or the inhibition of autophagosome clearance, could explain the increases observed. However, a decrease in autolysosome maturation indicates an inhibition of autophagosome clearance. LC3-II protein expression and autophagy puncta fold changes were assessed in the presence of modulators of the autophagy pathway to discriminate between these two possibilities. In the presence of chloroquine or bafilomycin a1, autophagic flux, as assessed by LC3-II expression changes by western blotting, was significantly decreased in *LRRK2-R1441C* primary cortical cultures (Fig. 2A and B). In order to assess if this altered autophagic flux was present in neurons in these cortical cultures, co-immunofluorescence for LC3 and LAMP1 was performed. The autolysosome count for *LRRK2-R1441C* neurons did not decrease with chloroquine as observed in the other three genotypes (Supplementary Material, Fig. S3a, b). Taken together, these data suggest a block in autophagosome clearance and a deficit in autolysosome maturation in *LRRK2-R1441C* neurons, as opposed to an increase in autophagy induction. Interestingly, both *hWT-LRRK2* and *LRRK2-G2019S* neurons demonstrate a significant decrease in autophagic flux as assessed by western blotting, which reflected a decrease in autophagosome fold-change relative to nTG with chloroquine treatment, with no differences seen in autolysosome fold-change as measured by co-immunofluorescence, indicating inhibited autophagosome biogenesis.

To assess whether the accumulation of autophagosomes in *LRRK2-R1441C* neurons could be rescued by increasing lysosome number, neuronal cultures were treated with 500 μm trehalose, a compound known to induce lysosomal biogenesis (28). Increased LAMP1 expression was observed with a 24-h trehalose treatment in all genotypes, indicating an increase in lysosomes (Supplementary Material, Fig. S3c). Both autophagic flux, as assessed by changes in LC3-II expression in the presence of trehalose and bafilomycin a1, and autolysosomal fold-change from basal levels remained significantly decreased in *LRRK2-R1441C* neurons compared to nTG (Fig. 2C–E), suggesting an inherent autophagosome–lysosome fusion deficiency in these neurons. Furthermore, there was a significant decrease in autophagosome fold-change from basal levels in all three *hLRRK2* genotypes compared to non-transgenic neurons, suggesting an inhibitory effect of hLRRK2 expression on autophagosome production.

### *LRRK2-R1441C* neuronal cultures exhibit decreases in lysosomal protein degradation

To assess whether decreases in autolysosome maturation were coupled with changes in lysosomal protein degradation, a pulse-chase assay using C^14^-radiolabelled valine was carried out based on the protocol developed by Gronostajski and Pardee (29). The data demonstrate that in *LRRK2-R1441C* neuronal cultures lysosomal protein degradation was significantly decreased (Fig. 2F). Furthermore, *hWT-LRRK2* and *LRRK2-G2019S* cultures had decreased lysosomal protein degradation compared to nTG controls at the final 24-h time point but were still significantly greater compared to *LRRK2-R1441C* cultures. These changes in protein degradation reflect the changes seen in autophagy puncta counts and autophagic flux already described and confirm that the presence of hWT-LRRK2 or LRRK2-G2019S inhibits the production of autophagosomes, whereas LRRK2-R1441C is capable of decreasing autolysosome maturation.

### The *LRRK2-R1441C* autophagy phenotype is not dependent on LRRK2 kinase activity

The overexpression of the LRRK2 kinase domain as well as inhibition of LRRK2 kinase activity induces alterations of the autophagy-lysosomal pathway (26,30). Primary cortical neuronal cultures were prepared and treated with 50 nm of the selective and potent LRRK2 inhibitors MLi-2 and PF-06447475 for 2 h, and LRRK2 phosphorylation levels were measured to confirm that kinase activity had been decreased (Fig. 3A). At basal conditions, an increase in phosphorylated LRRK2 at Ser1292 was observed in *LRRK2-G2019S* and *LRRK2-R1441C* cultures relative to *hWT-LRRK2* (Fig. 3B). LC3-II protein levels were assessed by western blot to observe potential downstream effects of LRRK2 kinase inhibition on autophagy. *hWT-LRRK2* and *LRRK2-G2019S* cultures both demonstrated increased autophagic flux in response to both MLi-2 and PF-06447475 treatment, while no significant changes were seen in *LRRK2-R1441C* cultures (Fig. 3C and D). Interestingly, the LRRK2 kinase inhibitors had the opposite effect on endogenous Lrrk2 in nTG cultures, decreasing autophagic flux. In order to confirm that observed alterations in LC3-II levels were dependent on LRRK2 kinase inhibition as opposed to off-target effects, *Lrrk2^−/−^* primary cortical neuronal cultures were treated with MLi-2. No changes in LC3-II levels were observed in *Lrrk2−/−* cultures with MLi-2 treatment relative to WT litter mate controls (Supplementary Material, Fig. S3e, f).

We next performed pulse-chase assays to quantify protein degradation in response to LRRK2 kinase inhibition. Lysosomal protein degradation was significantly increased in the presence of the LRRK2 kinase inhibitors in both *hWT-LRRK2* and *LRRK2-G2019S* cultures (Fig. 3E), correlating with the increase in LC3-II protein levels as measured by flux assays. *LRRK2-R1441C* cultures, however, showed no significant changes in protein degradation levels with kinase inhibition. Finally, a significant decrease in lysosomal protein degradation was also observed in nTG primary neuronal cultures with MLi-2 and PF-06447475 treatment correlated with the decrease in LC3-II protein expression.

### *LRRK2-R1441C* neurons have impaired lysosome pH

**Figure 4:**
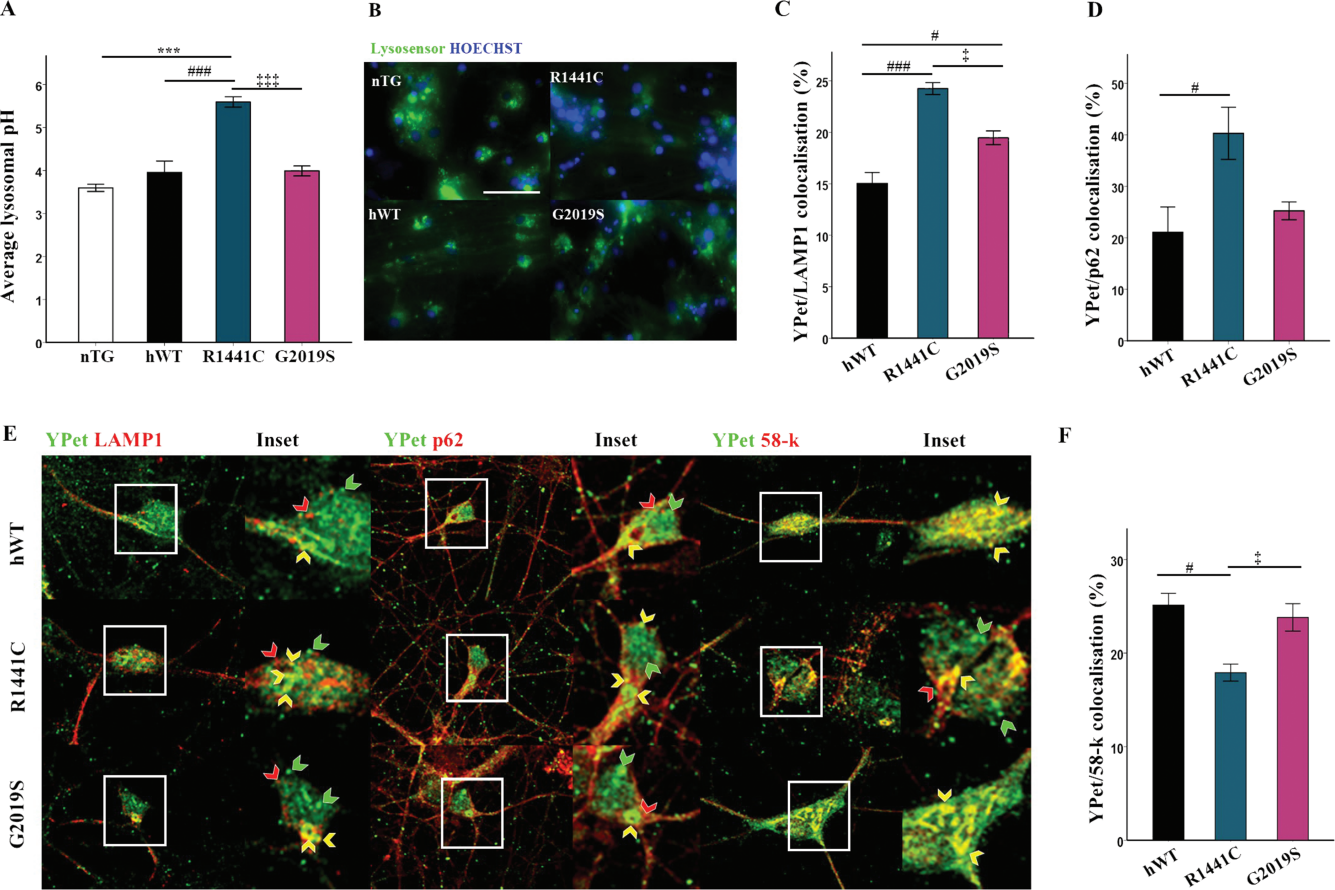
*LRRK2-R1441C* neuronal cultures exhibit increased lysosomal pH and co-localization with autophagic proteins. (**A, B**) Using the pH sensitive dye lysosensor lysosomal pH was quantified in primary cortical neuronal cultures from P1 pups at DIV 14. Bars, mean +/− SEM (N = 3; ^***^P < 0.001; Kruskal–Wallis non-parametric ANOVA, Bonferroni post hoc). Scale bar = 200 μm. (**C, D, E**) DIV 14 primary cortical neuronal cultures were fixed and stained for YPet and LAMP1/p62/58-k and co-localization quantified. Bars, mean +/− SEM (N = 3; ^*^P < 0.05; ^***^P < 0.001; one-way ANOVA, Bonferroni post hoc). (**F**) Representative confocal images of YPet and LAMP1/p62/58-k staining. Scale bar = 10 μm. Red arrow, LAMP1/p62/58-k only; green arrow, YPet only; yellow arrow, YPet co-localization with LAMP1/p62/58-k.

Both lysosomal degradation and autophagosome/lysosome fusion are highly dependent on lysosomal pH. As defects in autolysosome maturation were evident in *LRRK2-R1441C* neurons, we asked if altered lysosomal pH could account for the compromised vesicle fusion. Lysosomal pH was measured using the pH sensitive dye, LysoSensor™ Green DND-189, which exhibits increased fluorescence in acidic organelles, using a pH/fluorescence standard curve in cells incubated in pH-calibrated solutions (Supplementary Material, Fig. S3a). Measurement of average lysosomal pH across genotypes demonstrated that lysosomal pH was significantly increased in *LRRK2-R1441C* expressing cultures, with an average lysosomal pH of 5.5 (Fig. 4A and B).

### The *LRRK2-R1441C* mutations alters LRRK2 cellular localization

It has been reported that mutant and WT LRRK2 co-localize with markers of the autophagy pathway (23). Co-immunofluorescence staining in *LRRK2-R1441C* primary neurons demonstrated an increase in LRRK2-R1441C co-localization with the autophagy markers P62 and LAMP1 compared to both *hWT-LRRK2* and *LRRK2-G2019S* cultures (Fig. 4C–E; Supplementary Material, Fig. S4b). Interestingly, all transgenic neurons exhibited skein-like hLRRK2-positive structures revealed using the YPet tag as previously reported in cell lines harbouring the *LRRK2-R1441C* BAC constructs (23). Further co-immunofluorescence demonstrated that such skein-like structures show discrete co-localization to the trans-Golgi marker, 58 k (Fig. 4E; Supplementary Material, Fig. S4b). Furthermore, *LRRK2-R1441C* neurons exhibited decreased co-localization of hLRRK2 to the trans-Golgi marker compared to *hWT-LRRK2* and *LRRK2-G2019S* neurons (Fig. 4F). The decreased co-localization of LRRK2-R1441C to the trans-Golgi, and its increased co-localization to autophagic puncta, is a potential mechanism by which mutations alter hLRRK2 normal function.

### Increased autophagic flux and lysosomal protein degradation are observed in *Lrrk2^−/−^* neurons

Primary neuronal cultures were prepared from *Lrrk2* knockout (*Lrrk2^−/−^*) rats to compare the loss-of-function phenotype with mutant autophagy phenotypes likely caused by a gain of function. Lrrk2 expression in primary cortical cultures was first assessed to confirm knockout at the protein level (Supplementary Material, Fig. S5a). A significant increase in LC3-II protein levels was observed in *Lrrk2^−/−^* primary neurons compared to WT littermates (Supplementary Material, Fig. S5b) with no changes in LAMP1 or p62 protein expression observed (Supplementary Material, Fig. S5c, d). *Lrrk2^−/−^* primary cortical neuronal cultures and WT litter mate controls were treated with 20 μm chloroquine and LC3-II expression levels by western blotting were quantified in order to assess autophagic flux (Supplementary Material, Fig. S5e). A trend of increased autophagic flux was observed in *Lrrk2^−/−^* cultures, which did not reach significance (Supplementary Material, Fig. S5f)*.* However, using the more sensitive pulse-chase assay demonstrated that knock-out of the Lrrk2 protein was associated with an increase in lysosomal protein degradation (Supplementary Material, Fig. S5g). Overall, these data are consistent with WT Lrrk2 having a physiological function of reducing autophagic activity.

### *LRRK2-R1441C* neurons have impaired lysosome calcium dynamics

**Figure 5:**
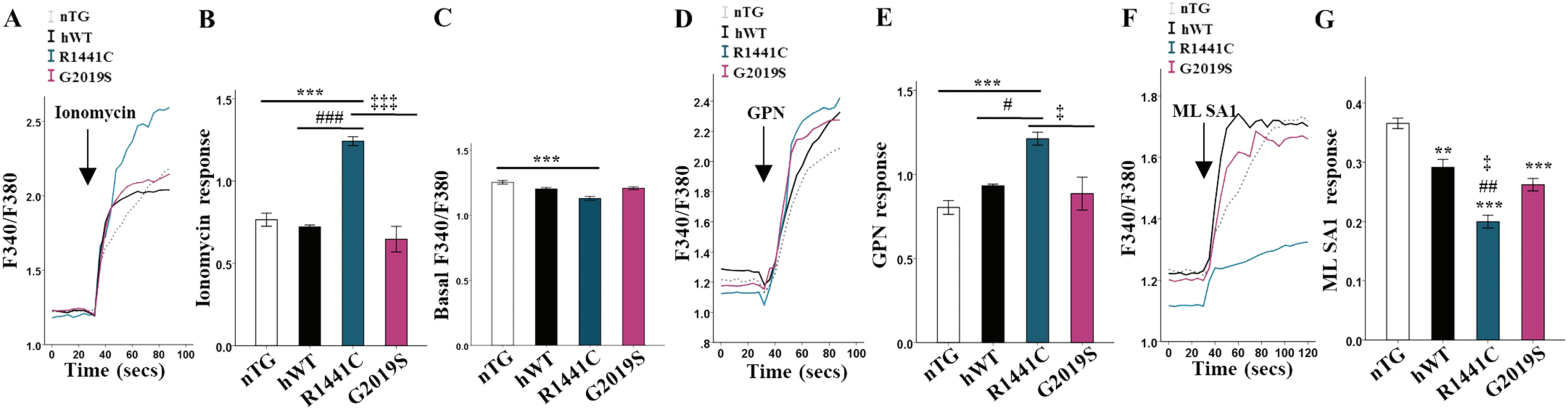
*LRRK2-R1441C* neurons have impaired lysosome calcium dynamics. (**A**, **B**) DIV 14 primary cortical neuronal cultures were incubated with the Fura2AM and fluorescence ratio quantified at basal and after the injection of ionomycin and delta quantified. (**C**) Baseline Fura2-AM was quantified across all lines to assess cytosolic Ca2+ content. (**D**, **E**) GPN was used in order to quantify lysosomal Ca2+ content. (**F, G**) ML SA1 was used in order to quantify Ca2+ release from TRPML1 channels.Bars, mean +/− SEM (N = 3; ^*^P < 0.05; ^**^P < 0.005; ^***^P < 0.001; Kruskal–Wallis non-parametric ANOVA, Bonferroni post hoc). ^*^, versus nTG; #, versus hWT; ‡, versus G2019S.

Local calcium release from lysosomes is required for late endosome–lysosome fusion (14). We therefore measured calcium signalling from different intracellular stores in *LRRK2-G2019S* and *LRRK2-R1441C* mutant primary neuronal cultures using the ratiometric calcium sensitive dye, Fura2-AM. We first showed a significantly increased whole-cell calcium content in *LRRK2-R1441C* cultures assayed by the response to ionomycin (Fig. 5A and B). It was also observed that LRRK2-R1441C expression induced a significantly decreased fluorescence ratio at basal levels prior to compound administration (Fig. 5C). Together this suggests an increase in intracellular calcium stores and a decrease in cytosolic calcium levels. We then administered compounds to assess the calcium content of specific organelles. The lysosome-disrupting compound, GPN, (Fig. 5D and E) led to an increased response in *LRRK2-R1441C* cultures. The increase in whole-cell calcium content in response to ionomycin was therefore likely caused by alterations in lysosomal calcium as no significant differences between genotypes in response to FCCP or CPA were observed (Supplementary Material, Fig. S6a, b, c, d) indicating no changes in mitochondria or ER calcium content, respectively.

The transient receptor potential cation channel, mucolipin subfamily, member 1 (TRPML1), is a cation channel located on lysosomal membranes and is one of the channels responsible for the local release of calcium. We used ML SA1, a compound capable of inducing TRPML1-mediated calcium release, to investigate release from lysosomes. We observed a significantly decreased lysosomal calcium release in response to ML SA1 in the *LRRK2-R1441C* cultures (Fig. 5F and G). Such decreased local calcium release may explain the decrease in fusion efficiency seen between the autophagosomes and lysosomes.

### LRRK2 interacts with vATPase a1 that is abolished by the *LRRK2-R1441C* mutation

**Figure 6:**
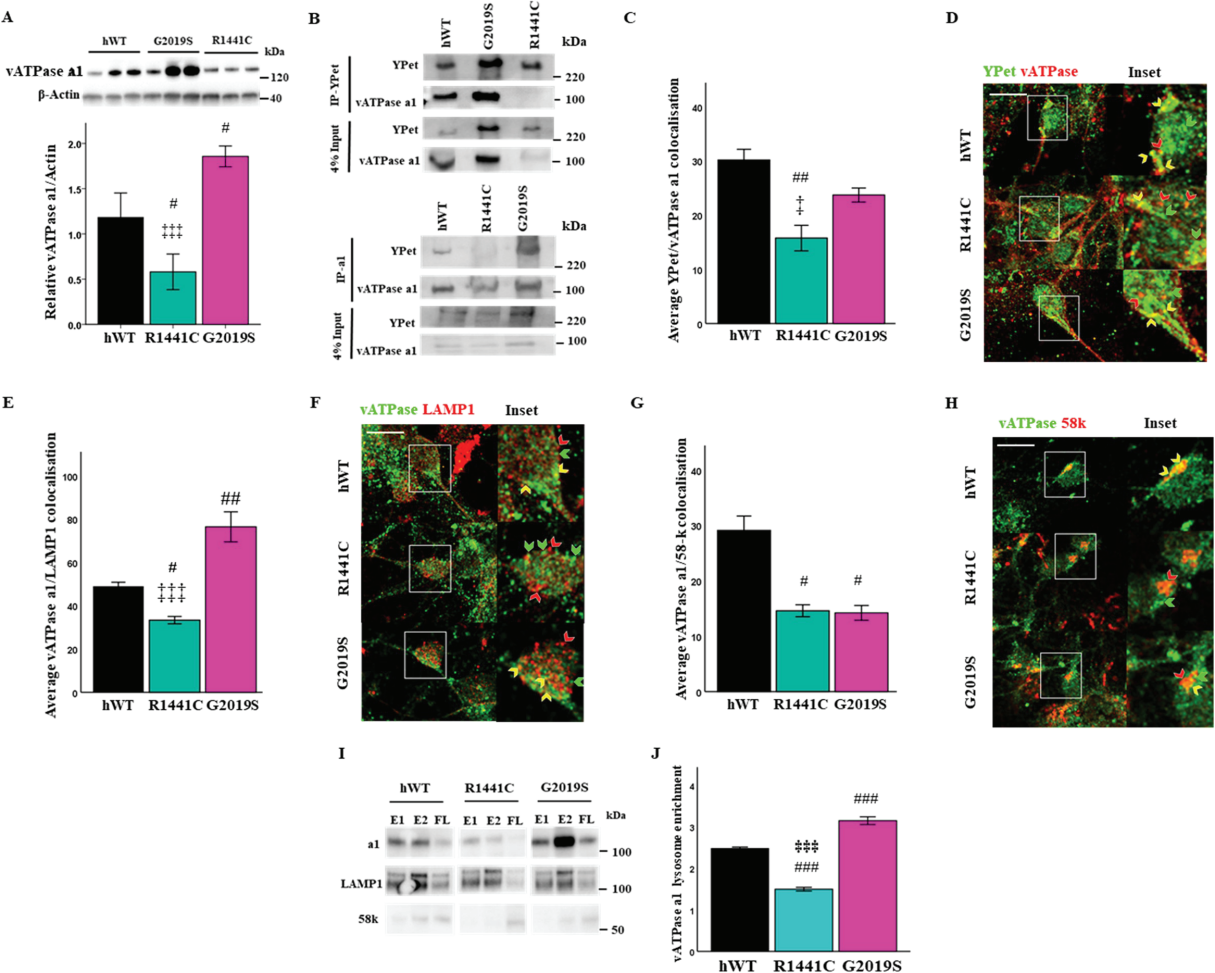
LRRK2 directly interacts with vATPase a1, which is abolished by the *LRRK2-R1441C* mutation. (**A**) Western blots for vATPase a1 were quantified to assess basal protein levels. Bars, mean +/− SEM (N = 3; ^*^P < 0.05; ^***^P < 0.001; Kruskal–Wallis non-parametric ANOVA, Bonferroni post hoc). (**B**) YPet and vATPase a1 were immunoprecipitated from LRRK2 transgenic primary cortical neuronal cultures at DIV 14 and vATPase a1/YPet protein expression observed. (**C, D**) DIV 14 primary cortical neuronal cultures were fixed and stained for YPet, vATPase a1 and β-III tubulin and co-localization percentage quantified. Co-localization between vATPase a1 and LAMP1 (**E, F**) or the Golgi marker 58-k (**G, H**) was quantified in DIV 14 cortical cultures. Scale bar = 10 μm. Arrows: red, red channel only; green, green channel only; yellow, co-localization of red and green channels. (**I, J**) Lysates from primary cortical neuronal cultures at DIV 14 were ultracentrifuged and lysosome-enriched fractions collected. vATPase a1, LAMP1 and 58-k protein expression were assessed in lysosome enriched fractions by western blot and quantified relative to flow-through (average densitometry value of E1 and E2 relative to FL; E1 = enriched fraction 1, E2 = enriched fraction 2, FL = flow-through). Bars, mean +/− SEM (N = 3; ^*^P < 0.05; ^**^P < 0.01; ^***^P < 0.001; one-way ANOVA, Bonferroni post hoc). #, versus hWT; ‡, versus G2019S.

The a1 subunit of the vATPase proton pump is enriched in neurons in the transmembrane V_0_ subcomplex of the vATPase, which is trafficked to and from the Golgi to the lysosome where it is able to form the full vATPase complex with the V_1_ subcomplex and regulate lysosomal pH levels. Protein level quantification revealed the a1 subunit to be downregulated in *LRRK2-R1441C* cultures and was also upregulated in *LRRK2-G2019S* cultures (Fig. 6A). Co-immunoprecipitation with YPet in hLRRK2-transgenic cell lysates was performed in order to assess binding to ATP6V0A1. hWT-LRRK2 and LRRK2-G2019S protein was observed to bind to this subunit; however, this interaction is abolished in the LRRK2-R1441C protein (Fig. 6B). nTG lysates were subject to co-immunoprecipitation with YPet in order to assess non-specific binding (Supplementary Material, Fig. S6e).

To confirm the observations in the co-immunoprecipitations, vATPase a1–YPet-tagged LRRK2 co-localization was assessed by co-immunofluorescence. A significant decrease in vATPase a1 co-localization to LRRK2-R1441C, relative to hWT-LRRK2, was observed (Fig. 6C and D; Supplementary Material, Fig. S6f). The vATPase a1 subunit is localized to the lysosomal membrane and is trafficked from the Golgi to its cellular target. The correct binding of vATPase a1 to interaction partners mediates this trafficking. vATPase a1 co-localization to both the Golgi and lysosomes was therefore assessed. A significant decrease in vATPase a1-LAMP1 co-localization was observed in *LRRK2-R1441C* neurons relative to both *hWT-LRRK2* and *LRRK2-G2019S*. Furthermore, a significant increase was observed in *LRRK2-G2019S* neurons, relative to *hWT-LRRK2* (Fig. 6E and F; Supplementary Material, Fig. S6f). A significant decrease in vATPase a1/58-k co-localization was also observed in both *LRRK2-R1441C* and *LRRK2-G2019S* neurons relative to *hWT-LRRK2* (Fig. 6G and H; Supplementary Material, Fig. S6f). vATPase a1 protein expression was assessed by western blotting in an enriched lysosome cell fraction to confirm this phenotype with a biochemical assay. Lysosome fractions were enriched for LAMP1 expression, while the trans-Golgi marker, 58-k, was reduced (Fig. 6I). vATPase a1 expression was significantly reduced in lysosome fractions from *LRRK2-R1441C* primary cortical neuronal cultures relative to hWT and *LRRK2-G2019S* cultures. Furthermore, *LRRK2-G2019S* primary cortical neuronal cultures demonstrated increased vATPase a1 expression in purified lysosome samples relative to *hWT-LRRK2* and *LRRK2-R1441C* (Fig. 6J). Overall, the loss of interaction between LRRK2-R1441C and vATPase a1 is associated with the mislocalization of vATPase a1. Furthermore, the increase in vATPase a1 protein expression in *LRRK2-G2019S* primary cortical neuronal cultures is associated with increased vATPase a1 localization to the lysosome in these neurons.

### Clioquinol rescues the *LRRK2-R1441C* cellular phenotypes

**Figure 7:**
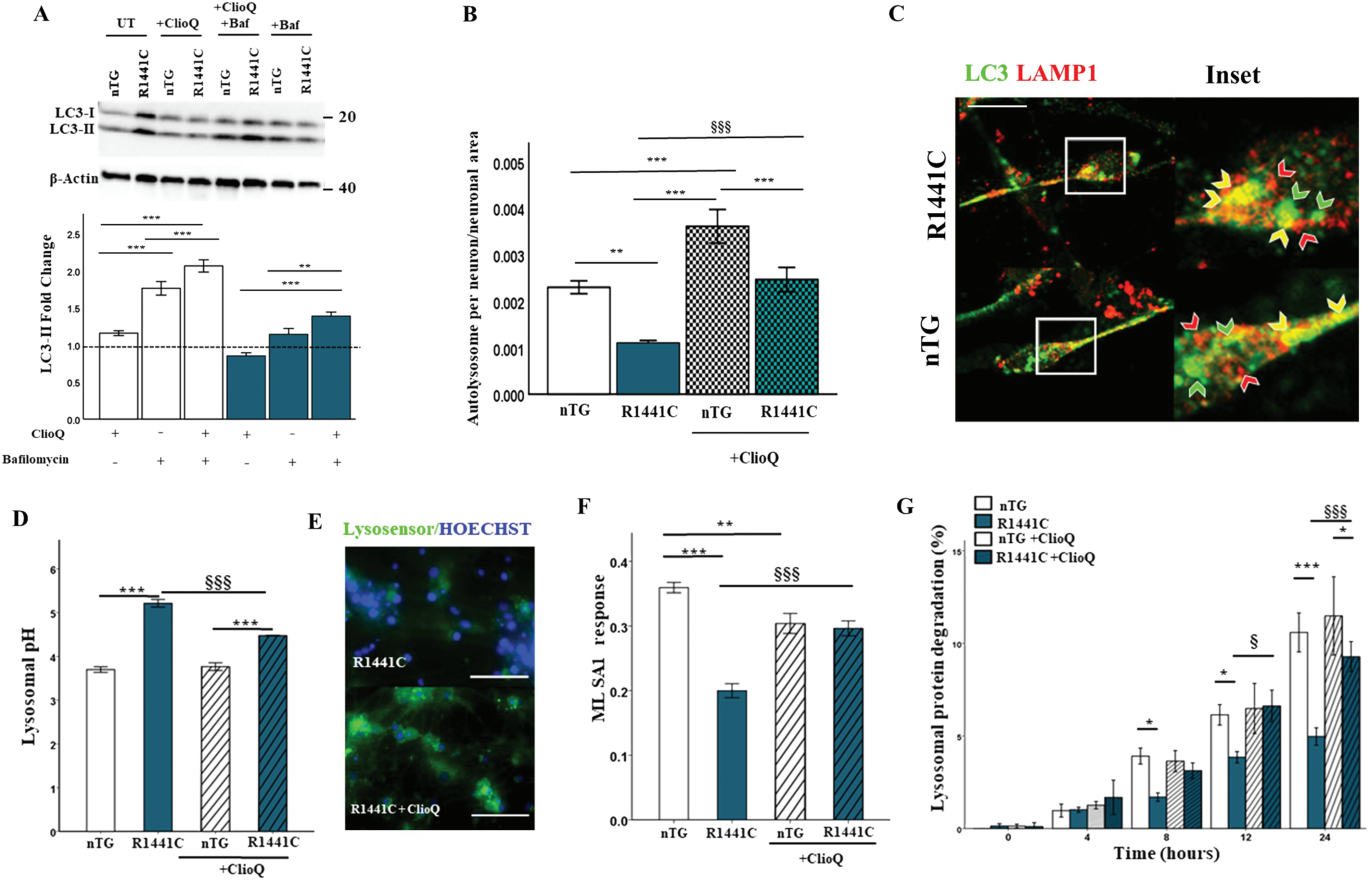
Clioquinol rescues autophagy related deficits in *LRRK2-R1441C* neuronal cultures. (**A**) DIV 14 primary cortical neuronal cultures were treated with 1 μm clioquinol for 48 h and/or 10 nm bafilomycin a1 for 2 h and LC3-II protein expression assessed to evaluate changes in autophagic flux. (**B, C**) DIV 14 primary cortical neuronal cultures were fixed and stained for LC3 and LAMP1 in β-III tubulin+ cells and autolysosomes/neuron normalized to neuronal size was quantified to assess changes in response to 1 μm clioquinol for 48 h. Scale bar = 5 μm. (**D, E**) Lysosomal pH was quantified in response to 1 μm clioquinol. Scale bar = 200 μm. (**F**) Primary cortical neuronal cultures were incubated in Fura2-AM and lysosomal calcium response to ML SA1 was measured either in the presence of or without 1 μm clioquinol pre-treatment. Bars, mean +/− SEM. (N = 3; ^*^P < 0.05; ^**^P < 0.01; ^***^P < 0.001; two-way ANOVA, Bonferroni post hoc)**.** (**G**) DIV 14 primary cortical neuronal cultures were incubated with radiolabelled valine for 48 h, and CPM was measured over 24 h in untreated cultures or in the presence of 1 μm clioquinol to calculate lysosomal protein degradation. Bars, mean +/− SEM (N = 3; ^*^P < 0.05; ^***^P < 0.001; three-way ANOVA, Bonferroni post hoc).^*^, versus nTG; §, versus R1441C.

It was recently reported that clioquinol, a zinc/copper ionophore, induces a cellular transcriptomic response anti-correlated to transcriptomic perturbations observed when comparing human *LRRK2-G2019S* with control induced pluripotent stem cell-derived dopamine neurons (31). To better understand the effect of clioquinol on the key LRRK2 mutant cellular phenotypes we observe in primary neurons, autophagic flux, autophagy puncta counts, lysosomal pH levels and protein-degradation assays were performed in the presence of the compound. Treatment of primary cortical neuronal cultures with clioquinol increased LC3-II flux in *LRRK2-R1441C* cultures (Fig. 7A) and significantly increased autolysosome counts in *LRRK2-R1441C* neurons (Fig. 7B and C; Supplementary Material, Fig. S7a, b). The *LRRK2-R1441C* lysosomal pH was also significantly re-acidified from basal levels in response to clioquinol (Fig. 7D and E). The presence of clioquinol increased lysosomal calcium release in *LRRK2-R1441C* primary cultures in response to ML SA1, indicating a correction of TRPML1 channel function (Fig. 7F; Supplementary Material, Fig. S7e), while total lysosomal calcium content increased (Supplementary Material, Fig. S7d, e). Furthermore, clioquinol was capable of increasing lysosomal protein degradation in *LRRK2-R1441C* cortical cultures as indicated by the pulse-chase assay (Fig. 7G).

### Clioquinol modulates lysosomal zinc levels and upregulates vATPase a1 protein

**Figure 8:**
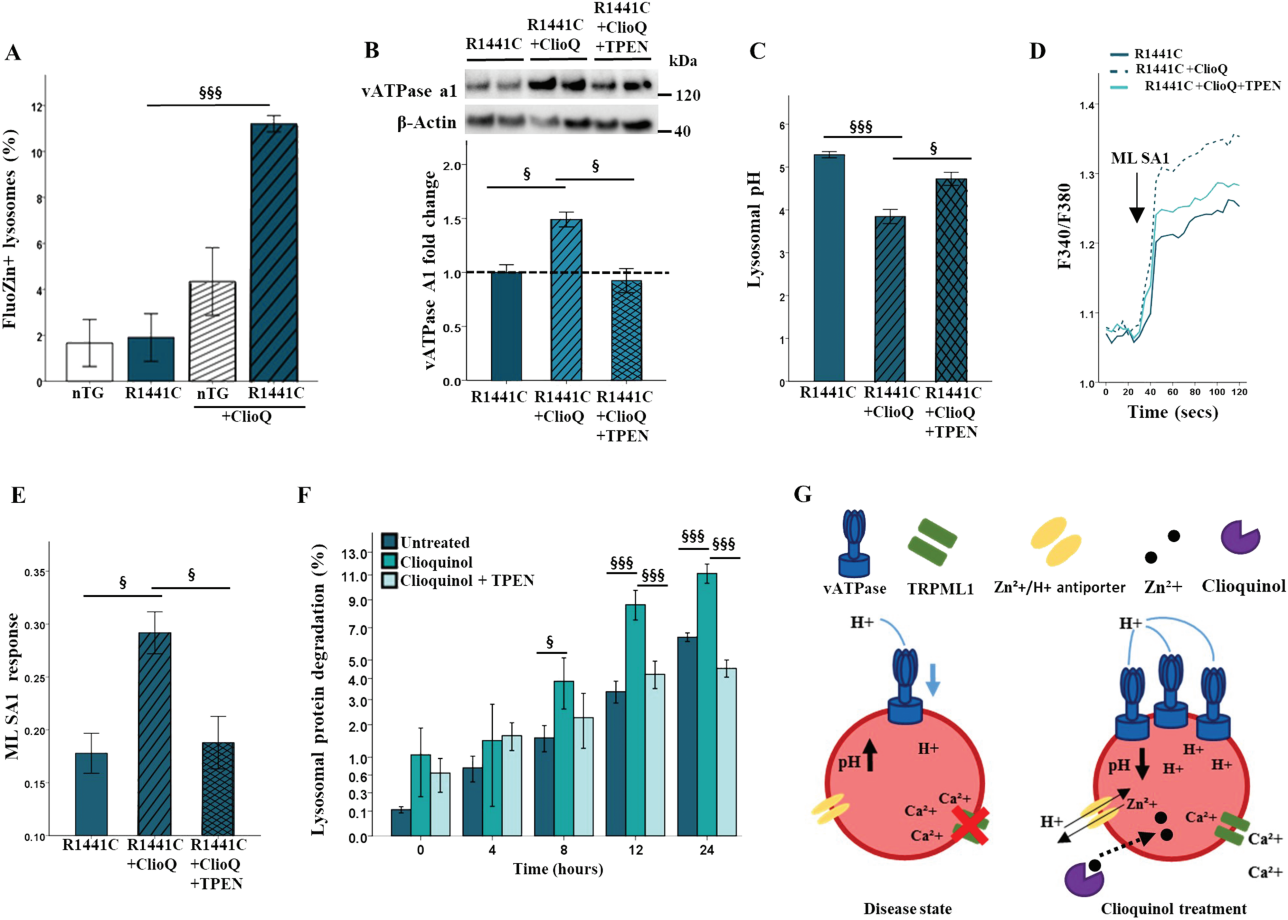
Clioquinol modulates lysosomal zinc levels and increase expression of vATPase a1. (**A, B**) Using the zinc-sensitive dye FluoZin-3 and the lysosome dye lysotracker, the percentage of FluoZin3+ lysosomes was quantified in the presence or absence of 1 μm clioquinol after 48 h. Bars, mean +/− SEM (N = 3; ^***^P < 0.001; two-way ANOVA, Bonferroni post hoc). (**B**) Western blots for vATPase a1 in *LRRK2-R1441C* primary neuronal cultures in the presence or absence of 1 μm clioquinol for 48 h and 500 nm TPEN for 6 h were quantified. (**C**) Using the pH sensitive dye lysosensor, lysosomal pH was quantified in *LRRK2-R1441C* primary cortical neuronal cultures treated with 1 μm clioquinol. (**D, E**) ML SA1 was used to quantify calcium release from TRPML1 channels after 1 μm clioquinol treatment. Bars, mean +/− SEM. (N = 3; ^*^P < 0.05, ^***^P < 0.001; one-way ANOVA, Tukey HSD post hoc). (**F**) DIV 14 *LRRK2-R1441C* primary cortical neuronal cultures were incubated with radiolabelled valine for 48 h and CPM was measured over 24 h in untreated cultures or in the presence of 500 nm TPEN and/or 1 μm clioquinol to calculate lysosomal protein degradation. Bars, mean +/− SEM (N = 3; ^*^P < 0.05; ^**^P < 0.005; ^***^P < 0.001; two-way ANOVA, Bonferroni posthoc, §, versus R1441C. (**G**) Schematic diagram of disease phenotype and effect of clioquinol treatment on the lysosome.

Previous reports have suggested that clioquinol may increase lysosomal zinc levels, which then act to modulate pH (32). We therefore used the zinc-sensitive dye, Furo-Zin3, to determine the effect of clioquinol on lysosomal zinc levels in *LRRK2-R1441C* primary cortical neuronal cultures. We observed that clioquinol treatment significantly increased Furo-Zin3-positive lysosomes in *LRRK2-R1441C*, but not nTG cultures (Fig. 8A; Supplementary Material, Fig. S7f). Treatment of cortical cultures with the zinc chelator N,N,N′,N′-tetrakis(2-pyridylmethyl)ethane-1,2-diamine (TPEN) decreased Furo-Zin3 fluorescence (Supplementary Material, Fig. S7g), confirming specificity of this dye. We next investigated if lysosomal zinc levels may functionally upregulate, or increase protein expression of, vATPase a1. Clioquinol treatment significantly upregulated protein expression of the a1 subunit of vATPase in *LRRK2-R1441C* primary cortical neuronal cultures (Fig. 8B), without changing lysosomal number as assayed by lysotracker (Supplementary Material, Fig. S7h).

In order to experimentally determine whether the effects of clioquinol of *LRRK2-R1441C* phenotype were zinc dependent, the clioquinol rescue assays previously described were repeated in the presence of the TPEN. In the presence of TPEN, clioquinol was no longer able to significantly increase vATPase a1 protein levels, nor decrease lysosomal pH from basal levels in *LRRK2-R1441C* cultures (Fig. 8B and C, Supplementary Material, Fig. S7i). Similarly, alterations in calcium release from TRPML1 channels were no longer increased with clioquinol treatment when co-treated with TPEN (Fig. 8D and E). Using the pulse-chase assay, in the presence of TPEN, lysosomal protein degradation decreased back to levels comparable to basal (Fig. 8F). Together these data provide strong evidence for the essential role of zinc in clioquinol rescue in *LRRK2-R1441C* neuronal cultures.

## Discussion

Our work has demonstrated differential effects on the autophagy/lysosomal pathway by the *LRRK2-R1441C* and *hWT-LRRK2* and *LRRK2-G2019S* variants in primary neuronal cultures. We have shown that the *LRRK2-R1441C* mutation prevents the normal binding of LRRK2 protein to the a1 subunit of vATPase, which leads to increased lysosomal pH and dysregulated lysosomal calcium dynamics through decreased a1 subunit protein levels and its mislocalization, inhibiting the maturation of autolysosomes. The *LRRK2-R1441C* phenotypes were shown to be independent of LRRK2 kinase activity. Furthermore, the *LRRK2-R1441C* autophagic deficits were not limited to primary cortical neurons, as increased LC3 expression was observed in both cortical neurons and dopamine neurons of the nigra in aged *LRRK2-R1441C* animals. These observations place the *LRRK2-R1441C* mutation as acting at the lysosome, specifically at the point of autophagosome–lysosome fusion. By modulating lysosomal zinc content and increasing vATPase subunit protein levels, clioquinol is capable of rescuing the observed autolysosome maturation deficit by correcting both pH and calcium release from TRMPL1 channels, providing a novel mechanism to correct lysosomal dysfunction in PD.

The kinase-dependent decrease in autophagic flux described here with hWT-LRRK2 and LRRK2-G1029S expression, combined with the increases observed in *Lrrk2^−/−^* neurons, suggests that the expression of either hWT-LRRK2 or LRRK2-G2019S inhibits the production of autophagosomes, a phenotype that has previously been reported (25,33,34). It has recently been reported that LRRK2 is able to inhibit the autophagy pathway by its interaction with Beclin1-containing class II PI3-kinase (25). Furthermore, this Beclin1 complex has been reported to be found to co-localize with trans-Golgi makers (35). The co-localization of hWT-LRRK2 and LRRK2-G2019S to the trans-Golgi could therefore underlie the inhibitory role of LRRK2 on the autophagy pathway demonstrated here. Many validated and potential LRRK2 targets are involved in pathways converging and diverging on the TGN. For example, Rab8 and Rab10 have been validated as true LRRK2 targets (36) and both of these GTPases have been implicated in biosynthetic trafficking from the TGN to the ER and plasma membrane (37,38).

Autophagy phenotypes were also replicated *in vivo*, with *LRRK2-R1441C* neurons in aged cortical and nigral tissue exhibiting increased LC3-positive puncta, demonstrating that alterations in autophagic activity induced by the *LRRK2-R1441C* mutation are not specific to cultured cortical neurons. Interestingly, although no significant changes in LC3 expression were observed in the cortex of *LRRK2-G2019S* animals, increased LC3 expression was observed in dopamine neurons of the substantia nigra pars compacta of these animals, which may contribute to the selective vulnerability of these dopamine neurons due to increased metabolic pressure (39). It has also been previously suggested that, although LRRK2 is ubiquitously expressed, LRRK2 is expressed at different levels in different brain regions (40). These differences, as well as the identification of specific functions of LRRK2 within specific systems such as the immune system (40,41), supports the hypothesis that LRRK2 could play different roles within different cell types and tissue.

*LRRK2-R1441C* neurons exhibited increased lysosomal pH, a critical phenotype for the decreased autolysosome maturation described. Substantial shifts in pH, such as those induced by drugs like chloroquine, severely disrupt degradative enzyme activity and also block fusion of autophagosomes and lysosomes (13). Elevations in lysosomal pH have previously been reported in cellular models in Alzheimer’s disease (42,43), and lysosome dysfunction is increasingly placed at the centre of neurodegenerative disease pathways. Increased lysosomal pH would not only decrease lysosomal fusion capacity and lysosomal enzyme activity, but has also been reported to have much more far-reaching consequences. Altered lysosomal degradation induces axonal transport impairment (45) and increases oxidized lipids and reactive oxygen species (45), leading to lysosomal membrane permeabilization resulting in apoptosis and necrosis (46).

The a1 subunit of the vATPase proton pump was found to be mislocalized in the presence of LRRK2-R1441C, presumably due to the loss in LRRK2-vATPase a1 interaction observed. Interestingly, in a recent meta-analysis of genome-wide association studies *ATP6V0A1,* encoding the vATPase a1 subunit was identified as a novel PD risk loci (19). This highlights not only the crucial role of the lysosome in PD but also the potential interplay between PD genetic risk factors. This same subunit has been shown to become destabilized in Presenilin-1 knock-out (PS1KO) cells, a model of Alzheimer’s disease, leading to elevated lysosomal pH (47). Furthermore, the blocking or knock-down of this sub-unit was capable of replicating the reported lysosomal phenotypes in PS1KO cells. The N-terminus of this subunit is essential for vATPase function because it serves to tether V1 to the membrane and must withstand the torque generated by V1 during motor movement (48). In addition, this region contains the information necessary for correct targeting of vATPases to cellular destinations (49). It is intriguing, therefore, that it is this subunit that LRRK2 interacts with and is downregulated in *LRRK2-R1441C* cultures. It has previously been demonstrated that modulation of vATPase a1 binding partners can negatively affect its function and lead to autolysosome maturation deficits similar to what has been described here (50). It has recently been shown that the membrane-anchored a1 subunit of the vATPase undergoes S-palmitoylation on Cys-25 and that expression is significantly decreased in palmitoyl-protein thioesterase 1 (Ppt1) deficient mice (51). Furthermore, dynamic palmitoylation (palmitoylation–depalmitoylation) facilitates the degradation of proteins that undergo palmitoylation (52). Therefore, alterations in vATPase a1 palmitoylation in *LRRK2-R1441C* cultures may underlie the decrease in vATPase a1 expression levels.

We have shown that LRRK2 kinase inhibition is able to ameliorate the inhibitory effect of hWT-LRRK2 and LRRK2-G2019S on autophagosome biogenesis, consistent with previous findings (25). However, kinase inhibitors had no effect on *LRRK2-R1441C* phenotypes, suggesting that kinase activity may not critical to the lysosomal phenotypes. It may be that the differential effects of the *LRRK2-G2019S* and *LRRK2-R1441C* mutations on lysosomal dysfunction may reside in differences in GTPase activity in these two mutations, and that the GTPase domain may be critical to the role of LRRK2 at the lysosome. Although the *LRRK2-G2019S* mutation consistently increases kinase activity (53,54,55,56), reports on the effect of other LRRK2 mutations residing in the kinase domain and in the GTPase domain on LRRK2-kinase activity have been inconsistent (4,7,53,55). Furthermore, mutants with hyperactive GTPase (R1298L) or inactive GTPase (R1398L/T1343 V) cannot mimic the G2019S phenotypes (57). While mutations in the GTPase domain have been reported to decrease GTP hydrolysis (58,59,60), the G2019S mutation has no discernible effects on GTPase activity (61). Taken together, it seems that mutations residing in different enzymatic domains exert different effects on the enzymatic activity of LRRK2, which impact cellular function in different ways. Understanding how LRRK2 GTPase activity contributes to cellular phenotypes is challenging and may require genetic or pharmacological manipulation of the GTPase domain.

Our work supports a mechanistic role for LRRK2 in calcium signalling in agreement with the effects on lysosomal calcium homeostasis previously reported (26). We demonstrate here that *LRRK2-R1441C* cultures exhibited a decrease in lysosomal calcium release from TRPML1 channels, which may in turn lead to the increase in lysosomal calcium content we observe. Interestingly, it has previously been reported that the opening probability and ion conductance of the TRPML1 channel is dependent on lysosomal pH, with increased pH decreasing function (62). It is therefore plausible that the primary deficit underlying the *LRRK2-R1441C* phenotype we describe here is a defective lysosomal pH, with alterations in lysosomal calcium release being secondary to this. Lysosomal pH is implicated in lysosomal calcium loading (63) with alkaline pH decreasing the activity of H+ pumps speculated as being responsible (64). However, it has recently been demonstrated that the inhibition of vATPase H+ pumps does not prevent lysosomal calcium refilling (65). It is therefore possible that the physiological mechanisms behind lysosomal calcium loading are not as fully understood as previously thought.

The zinc/copper ionophore, clioquinol, was capable of rescuing the *LRRK2-R1441C* phenotypes described, increasing autolysosome maturation, lysosomal protein degradation and decreasing lysosomal pH. Furthermore, we demonstrated that, with clioquinol treatment, the expression of the a1 subunit of v-ATPase significantly increased from its reduced basal levels. Using the zinc chelator TPEN, we further showed that the therapeutic role of clioquinol was dependent on its ability to mobilize zinc into the lysosome. It is noticeable that with clioquinol treatment, *LRRK2-R1441C* cultures demonstrate significantly higher increase in zinc containing lysosomes to nTGs. Patients with PD have been reported to exhibit increased levels of both zinc and manganese in serum and cerebrospinal fluid (66,67). Whether this exaggerated increase in lysosomal zinc levels is indicative of increased intracellular basal levels in *LRRK2-R1441C* cells remains to be determined. Nonetheless, a role of metal ions in neurodegenerative disease is becoming more apparent (68,69).

In summary, our data highlight specific novel phenotypes caused by *LRRK2* mutations and reveal further insight into normal physiological function of the WT protein. We demonstrate how two different mutations in different enzymatic cores of the same protein manifest in two very different phenotypes in the same cellular pathway, helping to ascribe a distinct function to the two enzymatic cores of LRRK2. We propose that the role of LRRK2 at the lysosome, specifically in regulating lysosomal pH and local calcium release by vATPase a1 binding and trafficking, is a crucial pathological mechanism linking autophagic deficits with Parkinson’s. The demonstration that small molecules that directly target lysosome dysfunction, such as clioquinol, have potential therapeutic benefit for PD fits closely with the emerging consensus from genetics on this critical area of cell biology.

## Materials and Methods

### Generation of primary cortical neurons

Transgenic male rats were bred with a pair of Sprague Dawley female to produce litters. P1 rats were tail clipped and marked for genotyping. A minimum of three animals per genotype were pooled for each experiment. Brains were extracted and meninges were removed, and cortical areas were dissected and shredded into 0.5 mm pieces. Tissue was incubated at 37°C in Neurobasal-A medium (Life Technologies) plus 0.01% trypsin and 0.5 μg/ml DNase for 20 min. Trypsin was inactivated with foetal bovine serum containing Neurobasal-A medium and samples centrifuged at 1000 rpm at 37°C for 5 min. This was then replaced with wash media and centrifuged at 1000 rpm at 37°C for 3 min. Wash media was removed and replaced with growth media (Neurobasal-A media, 1× Anti-Anti, 2 mm L-glutamine, 1× Anti-Anti, 1.75 μg Fungizone, 1× B27). Tissue was triturated, and cells were counted and diluted for plating. Cells were kept at 37°C, 5% CO2, 95% relative humidity and 50% of medium was replaced every 2 days. All assays were completed at DIV 14.

### Immunostaining

Cells were fixed in methanol previously cooled to −20°C and then washed with DPBS. Paraffin embedded sections, cut to 8 μm, were dewaxed and rehydrated through a series of alcohol. Cells/slides were blocked for 60 min at room temperature then incubated with primary over night at 4°C. Samples were then washed and incubated with secondary antibodies for 1 h at room temperature and were then washed and incubated in 1 μg/ml DAPI (Life Technologies). Coverslips were mounted onto Superfrost® plus (VWR) microscope slides using Fluorsave reagent (Millipore). Samples were imaged using an Invitrogen™ EVOS™ FL Auto cell imaging system at 60× magnification and a Nikon™ A1R™ confocal at 100× magnification. Unless stated, all immunostaining data was obtained from β-III tubulin/MAP2-positive cells.

### Co-localization imaging and analysis

A minimum of 60 neurons were imaged per experimental condition across three technical replicates. For image analysis, images were quantified using Cell ProfilerTM. Neurons were identified manually using β-III tubulin stain. Puncta (LC3/LAMPamp1/p62/YPet/58-k) were converted to greyscale and enhanced, suppressing background and using the Otsu method (to threshold brightness) and size exclusion, puncta were identified. Puncta were then overlaid onto each other to identify those co-localizing (see Supplementary Materials). The same pipeline was used for lysosensor and FluoZin-3AM/LysoTracker Red DND-99 imaging.

### Western blotting

Cells were lysed in RIPA with protease and phosphatase inhibitors. Samples were loaded onto 4–15% Criterion Tris-HCl polyacrylamide gels (BioRad). Electrophoresis was performed at 150 V for ~ 45 min, and proteins were transferred to a polyvinylidene membrane using a Trans-Blot Turbo Transfer System (BioRad). Membranes were blocked for 1 h at room temperate and incubated with primary antibody in blocking solution overnight at 4°C. Membranes were washed with TBS-T and incubated in horseradish peroxidase-conjugated secondary antibody (BioRad) in blocking solution for 1 h. Membranes were washed in TBS-T and developed using ECL solution (Millipore) and imaged on a Chemidoc Touch imaging system (Biorad) and quantified using ImageLab.

### Lysosensor imaging and quantification of lysosome pH

Lysosomal pH was measured using the LysoSensor™ Green DND-189 (Life Technologies). Briefly, primary cortical cultures were labelled with 100 nm LysoSensor™ Green DND-189 in growth media for 1 h and either immediately imaged or incubated for 10 min in pH-calibrated solutions. Nuclei were stained with Hoechst 33258 for 10 min, and cells were imaged using an Invitrogen™ EVOS™ FL Auto cell imaging system at 20× magnification. pH values were then calculated using a standard curve generated from the pH calibrations. pH-calibration solutions were generated in Hanks Buffered Salt solution (GIBCO, Life Technologies) containing 20 mm MES (4-Morpholineethanesulfonic acid), 110 mm KCl and 20 mm NaCl, supplemented with 30 μm nigericin and 15 μm monensin, sterile filtered and titrated to pH values of 3, 4, 5, 6 and 7.

### ^14^C-valine pulse-chase protein degradation assay

^14^C-valine was added to each well of cells for 48 h at 37°C in a humidified incubator with 5% CO2. Cells were then washed with fresh media and incubated for a further 24 h in the final wash. After 24 h, media was replaced with fresh media containing 1 mm^12^C-valine. A total of 10 μm chloroquine was added to control wells to ensure radioactive counts measured were due to lysosomal protein degradation. Aliquots of media were collected from each well at various time points and proteins precipitated by adding to ice-cold 20% trichloroacetic acid. Samples were centrifuged at 17000*g* for 10 min to pellet the proteins, and the supernatant was transferred to a scintillation vial and scintillation fluid added. At the end of the time course the remaining media was removed and the cells lysed by addition of 0.1 m sodium hydroxide for 5 min at 37°C. The lysate was transferred to a scintillation vial and scintillation fluid added. Counts were measured using a scintillation counter and protein degradation by autophagy calculated as follows: % = 100× (total counts for supernatant/total counts for supernatant and lysate). The % calculated for chloroquine treated samples were subtracted from those of untreated samples to ensure the final values were representative of lysosomal protein degradation.

### Calcium imaging

Cortical cultures were incubated with 2.5 mm Fura2-AM (Molecular Devices) in calcium-free HBSS (ThermoFisher) supplemented with 20 mm HEPES and 0.02% pluronic F-127 (ThermoFisher) for 1 h and imaged on a FlexStation 3 Multi-Mode Microplate Reader (Molecular Devices). Light excitation at 340 nm and 380 nm and measurement of detection at 510 nm allowed the ratio of unbound:bound calcium signal to be calculated (F340/F380). After establishing a baseline signal for 30 s, calcium compounds were injected into wells and the ratio measured for 60/120 s. Δ (F_1_—F_0_) was calculated to assess calcium store content.

### Co-immunoprecipitation

DynabeadsTM Protein G (Invitrogen) were washed in PBS +/+ using a magnetic rack (BioRad) and incubated with 40 μg of antibody/sample overnight at 4°C while rotating. Beads were washed in PBS +/+, and beads were then incubated with cell lysates (150 μg) for 3 days at 4°C while rotating. Flow through was removed for analysis and beads washed in PBS +/+ and boiled at 65°C in 3× Laemmli sample Buffer and assessed by SDS-PAGE.

### Zinc imaging

Cortical cultures were incubated with 5 μm FluoZin-3AM (Life Technologies) and 100 nm LysoTracker Red DND-99 (Life Technologies) for 1 h in neuronal growth medium supplemented with 0.02% pluronic acid F127 (Life Technologies). Cells were washed to remove unbound dye and incubated for 30 min to allow for the de-esterification of the dye. Nuclei were stained with Hoechst 33258 for 10 min and imaged on an Invitrogen™ EVOS™ FL Auto cell imaging system at 20× magnification.

### Statistics and data analysis

Data and statistical analyses were performed using SPSS. Data were analyzed by either one-way or two-way analysis of variance (ANOVA) or by *t*-test. In instances when data did not fit parametric assumptions, Kruskal–Wallis non-parametric ANOVA was used. Post hoc tests following ANOVAs were conducted using Tukey HSD or Bonferroni correction. Two-tailed levels of significance were used, and *P* < 0.05 was considered statistically significant.

## Supplementary Material

Supp_ddz088

## References

[1] von S., Bornschein B., Wick R., Bötzel K., Sampaio C., Poewe W., Oertel W., Siebert U., Berger K. and Dodel R. (2005) Prevalence and incidence of Parkinson's disease in Europe. Eur. Neuropsychopharmacol., 15, 473–490. 15963700 10.1016/j.euroneuro.2005.04.007

[2] Singleton A.B., Farrer M.J. and Bonifati V. (2013) The genetics of Parkinson's disease: progress and therapeutic implications. Mov. Disord., 28, 14–23. 23389780 10.1002/mds.25249 PMC3578399

[3] Nalls M.A., Pankratz N., Lill C.M., Do C.B., Hernandez D.G., Saad M., DeStefano A.L., Kara E., Bras J., Sharma M. et al. (2014) Large-scale meta-analysis of genome-wide association data identifies six new risk loci for Parkinson's disease. Nat. Genet., 46, 989–993. 25064009 10.1038/ng.3043 PMC4146673

[4] West A.B., Moore D.J., Choi C., Andrabi S.A., Li X., Dikeman D., Biskup S., Zhang Z., Lim K.L., Dawson V.L. and Dawson T.M. (2007) Parkinson's disease-associated mutations in LRRK2 link enhanced GTP-binding and kinase activities to neuronal toxicity. Hum. Mol. Genet., 16, 223–232. 17200152 10.1093/hmg/ddl471

[5] Greggio E., Jain S., Kingsbury A., Bandophadayay R., Lewis P., Kaganovich A., Van Der M.P., Beilina A., Blackinton J., Thomas K.J. et al. (2006) Kinase activity is required for the toxic effects of mutant LRRK2/dardarin. Neurobiol. Dis., 23, 329–341. 16750377 10.1016/j.nbd.2006.04.001

[6] Heo H., Park J., Kim C., Han B., Kim K. and Seol W. (2010) LRRK2 enhances oxidative stress-induced neurotoxicity via its kinase activity. Exp. Cell Res., 316, 649–656. 19769964 10.1016/j.yexcr.2009.09.014

[7] Lewis P.A., Greggio E., Beilina A., Jain S., Baker A. and Cookson M. (2007) The R1441C mutation of LRRK2 disrupts GTP hydrolysis. Biochem. Biophys. Res. Commun., 357, 668–671. 17442267 10.1016/j.bbrc.2007.04.006 PMC1939973

[8] Xiong Y., Yuan C., Chen R., Dawson T.M. and Dawson V.L. (2012) ArfGAP1 is a GTPase activating protein for LRRK2: reciprocal regulation of ArfGAP1 by LRRK2. J. Neurosci., 32, 3877–3886. 22423108 10.1523/JNEUROSCI.4566-11.2012 PMC3319331

[9] Sloan M., Alegre-Abarrategui. J., Potgieter D., Kaufmann A., Exley R., Deltheil T., Threlfell S., Connor-Robson N., Brimblecombe K., Wallings R. et al. (2016) LRRK2 BAC transgenic rats develop progressive, L-DOPA-responsive motor impairment, and deficits in dopamine circuit function. Hum. Mol. Genet., 25, 951–963. 26744332 10.1093/hmg/ddv628 PMC4754049

[10] Ramonet D., Daher J., Lin B., Stafa K., Kim J., Banerjee R., Westerlund M., Pletnikova O., Glauser L., Yang L. et al. (2011) Dopaminergic neuronal loss, reduced neurite complexity and autophagic abnormalities in transgenic mice expressing G2019S mutant LRRK2. PLoS One, 6, 1–15, e18568. 10.1371/journal.pone.0018568 PMC3071839 21494637

[11] Li Y., Liu W., Oo T., Wang L., Tang Y., Jackson-Lewis V., Zhou C., Geghman K., Bogdanov M., Przedborski S. et al. (2009) Mutant LRRK2 (R1441G) BAC transgenic mice recapitulate cardinal features of Parkinson's disease. Nat. Neurosci., 12, 826–828. 19503083 10.1038/nn.2349 PMC2845930

[12] Tong Y., Yamaguchi H., Giaime E., Boyle S., Kopan R., Kelleher R. and Shen J. (2010) Loss of leucine-rich repeat kinase 2 causes impairment of protein degradation pathways, accumulation of alpha-synuclein, and apoptotic cell death in aged mice. Proc. Natl. Acad. Sci. U. S. A., 107, 9879–9884. 20457918 10.1073/pnas.1004676107 PMC2906862

[13] Klionsky D.J., Elazar Z., Seglen P.O. and Rubinsztein D.C. (2008) Does bafilomycin A1 block the fusion of autophagosomes with lysosomes? Autophagy, 4, 849–950. 18758232 10.4161/auto.6845

[14] Pryor P.R., Mullock B.M., Bright N.A., Gray S.R. and Luzio J.P. (2000) The role of intraorganellar Ca(2+) in late endosome-lysosome heterotypic fusion and in the reformation of lysosomes from hybrid organelles. J. Cell Biol., 149, 1053–1062. 10831609 10.1083/jcb.149.5.1053 PMC2174832

[15] Korolchuk V.I., Saiki S., Lichtenberg M., Siddiqi F., Roberts E., Imarisio S., Jahreiss L., Sarkar S., Futter M., Menzies M. et al. (2011) Lysosomal positioning coordinates cellular nutrient responses. Nat. Cell Biol., 13, 453–460. 21394080 10.1038/ncb2204 PMC3071334

[16] Cuervo A.M. and Dice J.F. (2000) Age-related decline in chaperone-mediated autophagy. J. Biol. Chem., 275, 31505–31513. 10806201 10.1074/jbc.M002102200

[17] Miura E., Hasegawa T., Konno M., Suzuki M., Sugeno N., Fujikaken N., Geisler S., Tabuchi M., Oshima R., Kikuchi A. et al. (2014) VPS35 dysfunction impairs lysosomal degradation of α-synuclein and exacerbates neurotoxicity in a drosophila model of Parkinson's disease. Neurobiol. Dis., 71, 1–13. 25107340 10.1016/j.nbd.2014.07.014

[18] Zimprich A., Benet-Pages A., Struhal W., Graf E., Sebastian M., Haubenberger D., Spielberger S., Lichtner P., Klopp N., Wolf E. et al. (2011) A mutation in VPS35, encoding a subunit of the retromer complex, causes late-onset Parkinson disease. Am. J. Hum. Genet., 89, 168–175. 21763483 10.1016/j.ajhg.2011.06.008 PMC3135812

[19] Chang D., Nalls M.A., Hallgrimsdottir I.B., Hunkapiller J., van der M., Cai F., International Parkinson’s Disease Genomics Consortium and Me Research Team, Kerchner G., Ayalon A. et al. (2017) A meta-analysis of genome-wide association studies identifies 17 new Parkinson's disease risk loci. Nat. Genet., 49, 1511–1516. 28892059 10.1038/ng.3955 PMC5812477

[20] Lees A.J. and Singleton A.B. (2007) Clinical heterogeneity of ATP13A2 linked disease (Kufor-Rakeb) justifies a PARK designation. Neurology, 68, 1553–1554. 17485640 10.1212/01.wnl.0000265228.66664.f4

[21] S E., Nalls M., Aasly J., Aharon-Peretz J., Annesi G., Barbosa E., Bar-Shira A., Berg D., Bras J. et al. (2009) Multicenter analysis of glucocerebrosidase mutations in Parkinson's disease. N. Engl. J. Med., 361, 1651–1661. 19846850 10.1056/NEJMoa0901281 PMC2856322

[22] Do C.B., Tung J., Dorfman E., Kiefer A., Drabant E., Francke U., Mountain J., Goldman S., Tanner C., Langston J. et al. (2011) Web-based genome-wide association study identifies two novel loci and a substantial genetic component for Parkinson’s disease. PLoS Genetics, 7, 1–14, e1002141. 10.1371/journal.pgen.1002141 PMC3121750 21738487

[23] Alegre-Abarrategui J., Christian H., Lufino M., Mutihac R., Venda L., Ansorge O. and Wade-Martins R. (2009) LRRK2 regulates autophagic activity and localizes to specific membrane microdomains in a novel human genomic reporter cellular model. Hum. Mol. Genet., 18, 4022–4034. 19640926 10.1093/hmg/ddp346 PMC2758136

[24] Alegre-Abarrategui J. and Wade-Martins R. (2009) Parkinson’s disease, LRRK2 and the endocytic-autophagic pathway. Autophagy, 5, 1208–1210. 19770575 10.4161/auto.5.8.9894

[25] Manzoni C., Mamais A., Roosen D., Dihanich S., Soutar M., Plun-Favreau H., Bandopadhyay R., Hardy J., Tooze S., Cookson M. et al. (2016) mTOR independent regulation of macroautophagy by Leucine rich repeat kinase 2 via Beclin-1. Sci. Rep., 6, 1–10, 35106. 27731364 10.1038/srep35106 PMC5059726

[26] Gomez-Suaga P., Chruchill G.C., Patel S. and Hilfiker S. (2012) A link between LRRK2, autophagy and NAADP-mediated endolysosomal calcium signalling. Biochem Soc. Trans., 40, 1140–1146. 22988879 10.1042/BST20120138

[27] Tong Y., Giaime E., Yamaguchi H., Ichimura T., Liu Y., Si H., Cai H., Bonventre J. and Shen J. (2012) Loss of leucine-rich repeat kinase 2 causes age-dependent bi-phasic alterations of the autophagy pathway. Mol. Neurodegener., 7, 1–16. 22230652 10.1186/1750-1326-7-2 PMC3296570

[28] Sergin I., Evans T., Zhang X., Bhattacharya S., Stokes C., Song E., Ali S., Dehestani B., Holloway K., Micevych P. et al. (2017) Exploiting macrophage autophagy-lysosomal biogenesis as a therapy for atherosclerosis. Nat. Commun., 8, 1–20, doi:10.1038/ncomms15750. 28589926 10.1038/ncomms15750 PMC5467270

[29] Gronostajski R.M. and Pardee A.B. (1984) Protein degradation in 3T3 cells and tumorigenic transformed 3T3 cells. J. Cell Physiol., 119, 127–132. 6323489 10.1002/jcp.1041190120

[30] Manzoni C., Mamais A., Dihanich S., McGoldrick. P., Devine M., Zerle J., Kara E., Taanman J., Healy D., Marti-Masso J. et al. (2013) Pathogenic Parkinson’s disease mutations across the functional domains of LRRK2 alter the autophagic/lysosomal response to starvation. Biochem. Biophys. Res. Commun., 441, 862–866. 24211199 10.1016/j.bbrc.2013.10.159 PMC3858825

[31] Sandor C., Roberts P., Lang C., Heger A., Booth H., Vowles J., Witty L., Bowden R., Hu M., Cowle S. et al. (2017) Transcriptomic profiling of purified patient-derived dopamine neurons identifies convergent perturbations and therapeutics for Parkinson’s disease. Hum. Mol. Genet., 26, 552–566. 28096185 10.1093/hmg/ddw412 PMC5409122

[32] Seo B.R., Lee S.J., Cho K.S., Yoon Y.H. and Koh J.Y. (2015) The zinc ionophore clioquinol reverses autophagy arrest in chloroquine-treated ARPE-19 cells and in APP/mutant presenilin-1-transfected Chinese hamster ovary cells. Neurobiol. Aging., 36, 3228–3238. 26453000 10.1016/j.neurobiolaging.2015.09.006

[33] Sanchez-Danes A., Richaud-Patin Y., Carballo-Carbajal I., Jimenez-Delgado S., Caig C., Mora S., Di Guglielmo C., Ezquerra M., Patel B., Giralt A. et al. (2012) Disease-specific phenotypes in dopamine neurons from human iPS-based models of genetic and sporadic Parkinson's disease. EMBO Mol. Med., 4, 380–395. 22407749 10.1002/emmm.201200215 PMC3403296

[34] Su Y.C. and Qi X. (2013) Inhibition of excessive mitochondrial fission reduced aberrant autophagy and neuronal damage caused by LRRK2 G2019S mutation. Hum. Mol. Genet., 22, 4545–4561. 23813973 10.1093/hmg/ddt301

[35] Kihara A., Kabeya Y., Ohsumi Y. and Yoshimori T. (2001) Beclin-phosphatidylinositol 3-kinase complex functions at the trans-Golgi network. EMBO Rep., 2, 330–335. 11306555 10.1093/embo-reports/kve061 PMC1083858

[36] Steger M., Tonelli F., Ito G., Davies P., Trost M., Vetter M., Wachter S., Lorentzen E., Duddy G., Wilson S. et al. (2016) Phosphoproteomics reveals that Parkinson's disease kinase LRRK2 regulates a subset of Rab GTPases. Elife, 5, 1–28, e12813. 10.7554/eLife.12813 PMC4769169 26824392

[37] Stenmark H. (2009) Rab GTPases as coordinators of vesicle traffic. Nat. Rev. Mol. Cell Biol., 10, 513–525. 19603039 10.1038/nrm2728

[38] Galea G. and Simpson J.C. (2015) High-content analysis of Rab protein function at the ER-Golgi interface. Bioarchitecture, 5, 44–53. 26693811 10.1080/19490992.2015.1102826 PMC4910929

[39] Surmeier D.J., Obeso J.A. and Halliday G.M. (2017) Selective neuronal vulnerability in Parkinson disease. Nat. Rev., 18, 101–113. 10.1038/nrn.2016.178 PMC5564322 28104909

[40] Giesert F., Hofmann A., Burger A., Zerle J., Kloos K., Hafen U., Ernst L., Zhang J., Vogt-Weisenhorn D. and Wurst W. (2013) Expression analysis of Lrrk1, Lrrk2 and Lrrk2 splice variants in mice. PLoS One, 8, 1–15, e63778. 10.1371/journal.pone.0063778 PMC3651128 23675505

[41] Thévenet J., Pescini Gobert R., Hooft van Huijsduijnen R., Wiessner C. and Sagot Y.J. (2011) Regulation of LRRK2 expression points to a functional role in human monocyte maturation. PLoS One, 6, e21519. 21738687 10.1371/journal.pone.0021519 PMC3124520

[42] Coffey E.E., Beckel J.A., Laties A.M. and Mitchell C.H. (2014) Lysosomal alkalization and dysfunction in human fibroblasts with the Alzheimer's disease-linked presenilin 1 A246E mutation can be reversed with cAMP. Neuroscience, 263, 111–124. 24418614 10.1016/j.neuroscience.2014.01.001 PMC4028113

[43] Lee J., Yu W., Kumar A., Lee S., Mohan P., Peterhoff C., Wolfe D., Martinez-Vicente M., Massey A., Sovak G. et al. (2010) Lysosomal proteolysis and autophagy require Presenilin 1 and are disrupted by Alzheimer-related PS1 mutations. Cell, 141, 1146–1158. 20541250 10.1016/j.cell.2010.05.008 PMC3647462

[44] L S., Sato Y. and Nixon R.A. (2011) Lysosomal proteolysis inhibition selectively disrupts axonal transport of degradative organelles and causes an alzheimer's-like axonal dystrophy. J. Neurosci., 31, 7817–7830. 21613495 10.1523/JNEUROSCI.6412-10.2011 PMC3351137

[45] Yang J., Wu L., Tashino S., Onodera S. and Ikejima T. (2008) Reactive oxygen species and nitric oxide regulate mitochondria-dependent apoptosis and autophagy in evodiamine-treated human cervix carcinoma HeLa cells. Free Radic. Res., 42, 492–504. 18484413 10.1080/10715760802112791

[46] Guicciardi M.E., Marcel L. and Gregory G. (2004) Lysosomes in cell death. Oncogene, 23, 2881–2890. 15077151 10.1038/sj.onc.1207512

[47] Lee J., Kumar A., Sato Y., Mohan P., Kompella U. and Lloyd-Evans E. (2015) Presenilin 1 maintains lysosomal Ca2+ homeostasis by regulating vATPase-mediated lysosome acidification. Cell Rep., 12, 1430–1444. 26299959 10.1016/j.celrep.2015.07.050 PMC4558203

[48] Cotter K., Stransky L., McGuire C. and Forgac M. (2015) Recent insights into the structure, regulation and function of the V-ATPases. Trends Biochem. Sci., 40, 611–622. 26410601 10.1016/j.tibs.2015.08.005 PMC4589219

[49] Finnigan G.C., Cronan G., Park H., Srinivasan S., Quiocho F. and Stevens T. (2012) Sorting of the yeast vacuolar-type, proton-translocating ATPase enzyme complex (V-ATPase): identification of a necessary and sufficient golgi/endosomal retention signal in stv1p. J. Biol. Chem., 287, 19487–19500. 22496448 10.1074/jbc.M112.343814 PMC3365986

[50] Namkoong S., Lee K., Lee J., Park R., Lee E., Jang I. and Park J. (2015) The integral membrane protein ITM2A, a transcriptional target of PKA-CREB, regulates autophagic flux via interaction with the vacuolar ATPase. Autophagy, 11, 756–768. 25951193 10.1080/15548627.2015.1034412 PMC4509440

[51] Bagh M.B., Peng S., Chandra G., Zhang Z., Singh S., Pattabiraman N., Liu A. and Mukherjee A. (2017) Misrouting of v-ATPase subunit V0a1 dysregulates lysosomal acidification in a neurodegenerative lysosomal storage disease model. Nat. Commun., 8, 1–16, 14612. 28266544 10.1038/ncomms14612 PMC5344305

[52] Bijlmakers M.J. and Marsh M. (2003) The on-off story of protein palmitoylation. Trends Cell Biol., 13, 32–42. 12480338 10.1016/s0962-8924(02)00008-9

[53] Covy J.P. and Giasson B.I. (2009) Identification of compounds that inhibit the kinase activity of leucine-rich repeat kinase 2. Biochem. Biophys. Res. Commun, 378, 473–477. 19027715 10.1016/j.bbrc.2008.11.048 PMC2633649

[54] Luzon-Toro B., De La E., Delgado A., Perez-Tur J. and Hilficker S. (2007) Mechanistic insight into the dominant mode of the Parkinson's disease-associated G2019S LRRK2 mutation. Hum. Mol. Genet., 16, 2031–2039. 17584768 10.1093/hmg/ddm151

[55] West A.B., Morre D., Biskup S., Bugayenko A., Smith W., Ross C., Dawson V. and Dawson T. (2005) Parkinson’s disease-associated mutations in leucine-rich repeat kinase 2 augment kinase activity. Proc. Natl. Acad. Sci. U. S. A., 102, 16842–16847. 16269541 10.1073/pnas.0507360102 PMC1283829

[56] Anand V.S. and Braithwaite S. (2009) LRRK2 in Parkinson's disease: biochemical functions. FEBS J., 276, 6428–6435. 19804416 10.1111/j.1742-4658.2009.07341.x

[57] Biosa A., Trancikova A., Civiero L., Glauser L., Bubacco L., Greggio E. and Moore E. (2013) GTPase activity regulates kinase activity and cellular phenotypes of Parkinson’s disease-associated LRRK2. Hum. Mol. Genet., 22, 1140–1156. 23241358 10.1093/hmg/dds522

[58] Li X., Tan Y., Poulose S., Olanow C., Huang X. and Yue Z. (2007) Leucine-rich repeat kinase 2 (LRRK2)/ PARK8 possesses GTPase activity that is altered in familial Parkinson’s disease R1441C/G mutants. J. Neurochem., 103, 238–247. 17623048 10.1111/j.1471-4159.2007.04743.x PMC2827244

[59] Daniëls V., Vancraenenbroeck R., Law B., Greggio E., Lobbestael E., Gao F., De Maeyer M., Cookson M., Harvey K., Baekelandt V. et al. (2011) Insight into the mode of action of the Lrrk2 Y1699C pathogenic mutant. J. Neurochem., 116, 304–315. 21073465 10.1111/j.1471-4159.2010.07105.x PMC3005098

[60] Liao J., Wu C., Burlak C., Zhang S., Sahm H., Wang M., Zhang Z., Vogel K., Federici M., Riddle S. et al. (2014) Parkinson disease-associated mutation R1441H in LRRK2 prolongs the `active state’ of its GTPase domain. Proc. Natl. Acad. Sci. U. S. A., 111, 4055–4060. 24591621 10.1073/pnas.1323285111 PMC3964117

[61] Xiong Y., Coombes C., Kilaru A., Li X., Gitler A., Bowers W., Dawson V., Dawson T. and Moore D. (2010) GTPase activity plays a key role in the pathobiology of LRRK2. PLoS Genet, 6, 1–18, e1000902. 10.1371/journal.pgen.1000902 PMC2851569 20386743

[62] Li M., Zhang W., Benvin N., Zhou X., Su D., Li H., Wang S., Michailidis L., Tong L., Li X. et al. (2017) Structural basis of dual Ca2+/pH regulation of the endolysosomal TRPML1 channel. Nat. Struct. Mol. Biol., 24, 205–213. 28112729 10.1038/nsmb.3362 PMC5336481

[63] Christensen K.A., Myers J.T. and Swanson J.A. (2002) pH-dependent regulation of lysosomal calcium in macrophages. J. Cell Sci., 115, 599–607. 11861766 10.1242/jcs.115.3.599

[64] Churchill G., Okada Y., Thomas J., Genazzani A., Patel S. and Galione A. (2002) NAADP mobilizes Ca(2+) from reserve granules, lysosome-related organelles, in sea urchin eggs. Cell, 111, 703–708. 12464181 10.1016/s0092-8674(02)01082-6

[65] Garrity A.G., Wang W., Collier C., Levey S., Gao Q. and Xu H. (2016) The endoplasmic reticulum, not the pH gradient, drives calcium refilling of lysosomes. Elife, 5, 1–6, e15887. 10.7554/eLife.15887 PMC4909396 27213518

[66] Fukushima T., Tan X. and Kanda H. (2011) Serum vitamins and heavy metals in blood and urine, and the correlations among them in Parkinson's disease patients in China. Neuroepidemiology, 36, 240–244. 21677448 10.1159/000328253

[67] Hozumi I., Hasegawa T., Honda A., Ozawa K., Hayashi Y., Hashimoto M., Yamada M., Koumura A., Sakurai T., Kimura A. et al. (2011) Patterns of levels of biological metals in CSF differ among neurodegenerative diseases. J. Neurol. Sci., 303, 95–99. 21292280 10.1016/j.jns.2011.01.003

[68] Sastre M., Ritchie C.W. and Nabil H. (2015) Metal ions in Alzheimer’s disease brain. JSM Alzheimer’s Dis. Related Dementia, 2, 1014.

[69] Ward R.J., Dexter D.T. and Crichton R.R. (2015) Neurodegenerative diseases and therapeutic strategies using iron chelators. J. Trace Elem. Med. Biol., 31, 267–273. 25716300 10.1016/j.jtemb.2014.12.012

