# Supplementary material for: LRRK2 interacts with the vacuolar-type H+-ATPase pump a1 subunit to regulate lysosomal function
Source: Hum Mol Genet. 2019 Apr 30;28(16):2696–710. doi: 10.1093/hmg/ddz088 (PMC6687951; doi:10.1093/hmg/ddz088)
Supplement: Supp_ddz088 [file supp_ddz088.zip › Supplementary figure legends.docx]

**Supplementary Figure 1.**  (**A, B, C**) Primary cortical neuronal cultures from P1 pups were generated and characterised. Western blots for LRRK2, LAMP1 and p62 were quantified and normalised to nTG littermate controls to assess total protein level (bars represent mean +/- SEM, N = 3; **, P<0.005; One-Way ANOVA, Tukey HSD post-hoc). (**D, E**) Cultures at DIV 14 were stained for MAP2, GFAP and NeuN in order to assess neuronal:non-neuronal cell ratio. Scale bar = 200 μm. (bars represent mean +/- SEM. N = 3; Kruskal-Wallis non-parametric ANOVA, Bonferroni post-hoc). (**F**) 14 DIV primary cortical neuronal cultures were fixed and stained for β-III tubulin, LC3 and LAMP1. (**G**) Lysosome size in pixel area was quantified from confocal images of LC3/LAMP1 staining in β-III tubulin+ cells. Bars represent +/- SEM. N = 3.

**Supplementary Figure 2.**  (**A**) 22 month old rat cortical slices were stained for LC3 and MAP2 and (**B, C**) LC3 puncta number normalised to cell size and puncta size were quantified. (**D**) 22 month old rat midbrain slices were stained for LC3 and TH and (**E, F**) LC3 puncta number normalised to cell size and puncta size were quantified. Bars represent mean +/- SEM. (N = 3; *, P<0.05; **, P<0.005; ***, P<0.001; one-way ANOVA, Tukey HSD post-hoc). * = vs. nTG, # = vs. hWT, ‡ = vs. G2019S. Scale bar = 50 μm.

**Supplementary Figure 3.** (**A, B**) 14 DIV primary cortical neuronal cultures were treated with 20 µM chloroquine for 8h, fixed and stained for LC3 and LAMP1 and puncta/neuron counts normalised to neuronal size to quantify fold changes from basal. Bars represent mean +/- SEM (N = 3; *, P < 0.05; **, P<0.005; ***, P<0.001; Kruskal-Wallis non-parametric ANOVA, bonferroni post-hoc). * = vs. nTG, # = vs. hWT, ‡ = vs. G2019S. Scale bar = 5 μm. Arrows: Red = lysosome, Yellow = Autolysosome, Green = Autophagosome. (**C**) 14 DIV primary cortical neuronal cultures were treated with 500 µM trehalose for 24h and LAMP1 protein expression assessed by western blotting. (**D**) 14 DIV primary cortical neuronal cultures were treated with 500 µM trehalose for 24h, fixed and stained for LC3 and LAMP1 and β-tubulin. (**E, F**) The LC3-II fold change in response to 50 nM MLi-2 for 1 hour was quantified in *Lrrk2-/-* DIV 14 primary cortical neuronal cultures and WT litter mate controls (Bars represent mean +/- SEM, N = 3; Independent Samples *t*-test).

**Supplementary Figure 4.** (**A**) Using the pH sensitive dye Lysosensor pH titrated buffers were used to quantify a lysosomal pH standard curve. (**B**) Representative confocal images of YPet colocalisation with LAMP1/P62/58-k. Scale bar = 10 μm.

**Supplementary Figure 5.** (**A**) Primary cortical neuronal cultures from P1 pups were generated and characterised. Western blots for Lrrk2 were quantified to ensure knock-out. (**B, C, D**) Western blots for LC3-II, p62 and LAMP1 were quantified and normalised to nTG littermate controls in order to assess basal protein levels. (**E, F**) *Lrrk2-/-* DIV 14 primary neuronal cultures and litter mate controls were treated with 20 µM chloroquine for 8h and LC3-II expression levels quantified to assess autophagic flux. Bars represent mean +/- SEM. N = 3; *, P<0.05; Independent Samples *t*-test. (**G**) 14 DIV primary cortical neuronal cultures were incubated with radiolabelled valine for 48 hours and CPM was measured over 24 hours in untreated cultures or in the presence of chloroquine to calculate lysosomal protein degradation. Bars represent mean +/- SEM (N = 3; *, P < 0.05; Two-way ANOVA, Bonferroni post-hoc).

**Supplementary Figure 6.** (**A, B, C, D**) 14 DIV primary cortical neuronal cultures were incubated with the Fura2-AM and fluorescence ratio quantified at basal and after the injection of CPA/FCCP and delta quantified. Bars represent mean +/- SEM. N = 3. Kruskal-Wallis non-parametric ANOVA, Bonferroni post-hoc. (**E**) nTG cortical culture lysates were co-immunoprecipitated for YPet and Lrrk2 and vATPase a1 protein expression assessed in input, flow-through and immunoprecipitation sample to assess non-specific binding of YPet antibody. (**F**) 14 DIV cortical cultures were fixed and stained for YPet/LAMP1/58k and vATPase a1 and β-III tubulin.

**Supplementary Figure 7.** (**A, B**) 14 DIV primary cortical neuronal cultures were fixed and stained for LC3 and LAMP1 in β-III tubulin+ cells and autophagy puncta/neuron normalised to neuronal size was quantified to assess changes in response to 1 µM clioquinol. Dotted line = basal puncta per neuron. Scale bar = 5 μm. Arrows: Red = Lysosomes, Yellow = Autolysosomes, Grenn = Autophagosomes. Bars represent mean +/- SEM (N = 3; Kruskal-Wallis non-parametric ANOVA, Bonferroni post-hoc). * = vs. nTG, § = vs. R1441C. (**C**) Primary neuronal cultures were incubated in Fura2-AM and lysosomal calcium response to ML SA1 was measured either in the presence of or without 1 µM clioquinol pre-treatment. (**D, E**) Cortical cultures were incubated in Fura2-AM and lysosomal calcium response to GPN was measured either in the presence or without clioquinol pre-treatment. Bars represent mean +/- SEM (N = 3; *, P < 0.05; **, P < 0.01; ***, P < 0.001; Two-way ANOVA, Bonferroni post-hoc). (**F**) Representative images of FluoZin-3/Lysotracker live imaging. Scale bar = 200 µM. (**G**) 14 DIV primary neuronal cultures were incubated with FluoZin-3 in the presence or absence of 500 nM TPEN to assess for non-zinc-specific fluorescence. Scale bar = 200 µM.

(**H**) Lysosome number was quantified in *LRRK2-R1441C* cells with and without 48h 1 µM clioquinol treatment using Lysotracker (bars represent mean +/- SEM; N = 3; Independent Samples *t*-test). (**I**) Representative images of Lysosensor live imaging. Scale bar = 200 μm.
