# Supplementary material for: LRRK2 interacts with the vacuolar-type H+-ATPase pump a1 subunit to regulate lysosomal function
Source: Hum Mol Genet. 2019 Apr 30;28(16):2696–710. doi: 10.1093/hmg/ddz088 (PMC6687951; doi:10.1093/hmg/ddz088)
Supplement: Supp_ddz088 [file supp_ddz088.zip › Supplementary material.docx]

**Supplementary material**

**Animal husbandry**

Transgenic animals on a Sprague Dawley (SD) background were maintained in accordance with the UK Home Office regulations, under the Animals (Scientific Procedures) Act of 1986. Animals were caged in groups of 3-4 and had constant access to food and water. Room atmosphere was maintained at 22°C at 60-70% humidity and animals were kept in a 12 hour light/dark cycle.

**Genotyping**

Ear or tail clips were incubated in lysis buffer (50 mM KCl, 1.5 mM MgCl2, 10 mM Tris, 0.45% NP40, 0.45% Tween-20 and 0.5 mg/ml proteinase K, pH 8.5) for 2 hours at 55°C and at 95°C for 20 minutes to deactivate proteinase K. PCR was performed for the purposes of genotyping (genomic DNA, 1 x PCR buffer, 1 mM forward and reverse primer, MgCl2, Q-solution, 100 μM dNTPs, Ampli Taq-Gold DNA-polymerase). Human specific primers were designed for exons 8, 20 and 38 of the *LRRK2* transgene.

**Primary cortical culture treatments**

| **Compound** | **Supplier** | **Catalogue #** | **Concentration** | **Treatment time** |
| --- | --- | --- | --- | --- |
| Chloroquine | Sigma | C6628 | 20 µM | 8 h |
| Trehalose | Sigma | T9531 | 500 µM | 24 h |
| Clioquinol | Abcam | ab120355 | 1 µM | 48 h |
| Ionomycin | Sigma | I0634 | 8 µM | - |
| GPN | Abcam | ab145914 | 1.5 mM | - |
| CPA | Sigma | C1530 | 40 µM | - |
| FCCP | Sigma | C2920 | 80 µM | - |
| ML SA1 | Sigma | SML0627 | 1 mM | - |
| MLi-2 | Tocris | 1627091-47-7 | 50 nM | 1 h |
| PF-06447475 | Sigma | 1527473-33-1‎ | 50 nM | 1 h |
| TPEN | Sigma | P4413 | 500 nM | 6 h |
| Bafilomycin A1 | Sigma | B1793 | 10 nM | 2 h |

**Antibodies**

***Immunostaining***

| **Target** | **Supplier** | **Catalogue #** | **Species** | **Dilution** |
| --- | --- | --- | --- | --- |
| **B-III Tubulin** | Abcam | ab107216 | Chicken | 1:1000 |
| **GFAP** | Abcam | ab4674 | Chicken | 1:1000 |
| **MAP2** | Abcam | ab92434 | Chicken | 1:1000 |
| **MAP2** | Millipore | MAB5622 | Rabbit | 1:1000 |
| **NeuN** | Millipore | MAB5326 | Mouse | 1:500 |
| **LC3** | Cell Signalling | 2775 | Rabbit | 1:200 |
| **LAMP1** | Santa-cruz | Sc-20011 | Mouse | 1:100 |
| **GFP** | Invitrogen | A11122 | Rabbit | 1:100 |
| **LC3** | Sigma | SAB1305552 | Mouse | 1:200 |
| **P62** | Abcam | ab56416 | Mouse | 1:500 |
| **58k-Golgi** | Abcam | Ab27043 | Mouse | 1:500 |

***Western Blotting***

| **Target** | **Supplier** | **Catalogue** | **Species** | **Dilution** |
| --- | --- | --- | --- | --- |
| **Actin** | Abcam | ab49900 | Mouse | 1:50000 |
| **LC3** | Sigma | L7543 | Rabbit | 1:3000 |
| **Lamp1** | Abcam | ab24170 | Rabbit | 1:500 |
| **P62** | Abcam | Ab109012 | Rabbit | 1:1000 |
| **LRRK2** | Abcam | ab133474 | Rabbit | 1:500 |
| **GFP** | Invitrogen | A11122 | Rabbit | 1:100 |
