# Supplementary figures and images for: LRRK2 interacts with the vacuolar-type H+-ATPase pump a1 subunit to regulate lysosomal function
Source: Hum Mol Genet. 2019 Apr 30;28(16):2696–710. doi: 10.1093/hmg/ddz088 (PMC6687951; doi:10.1093/hmg/ddz088)

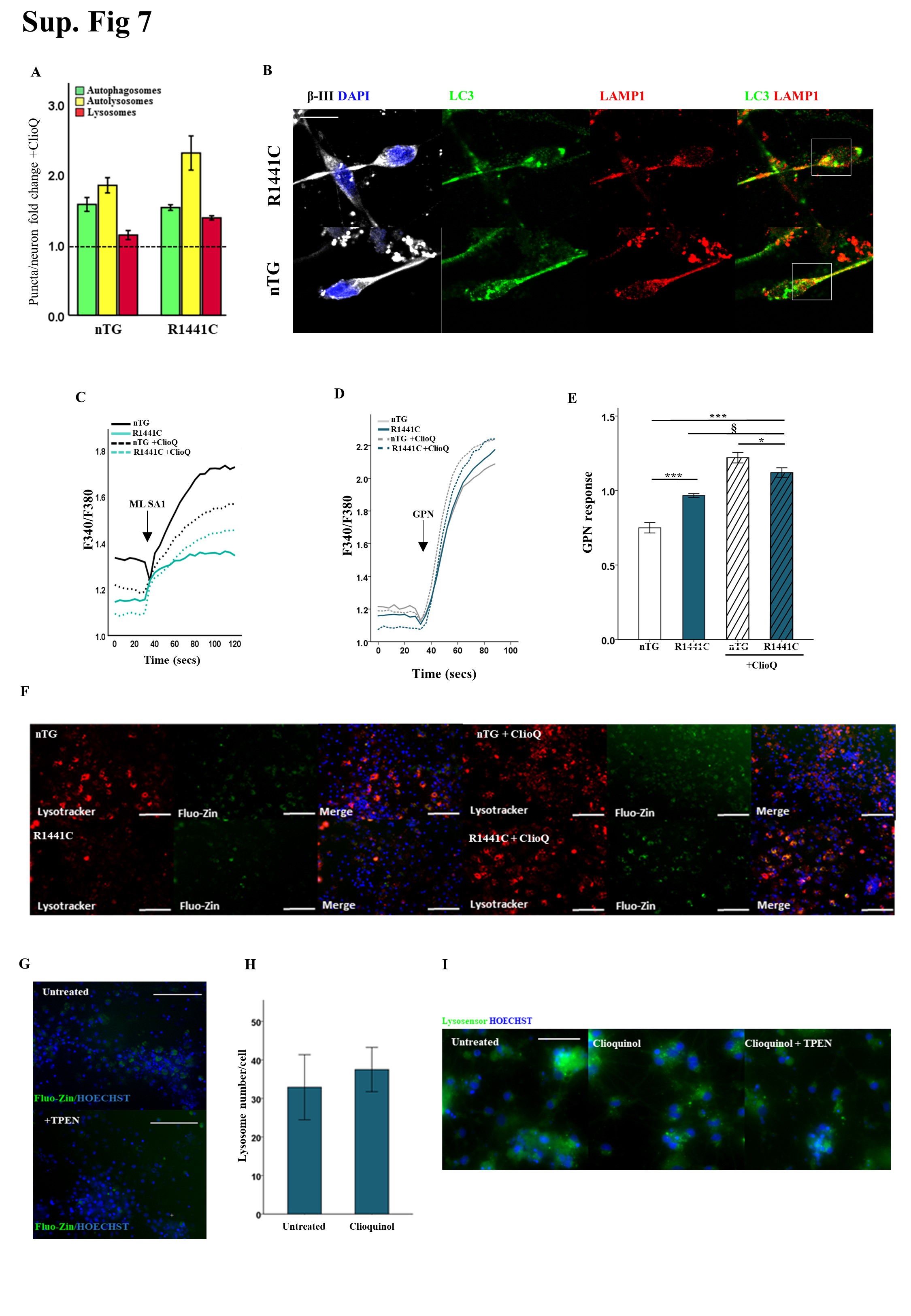

Supplement: Supp_ddz088 [file supp_ddz088.zip › Slide15.JPG]

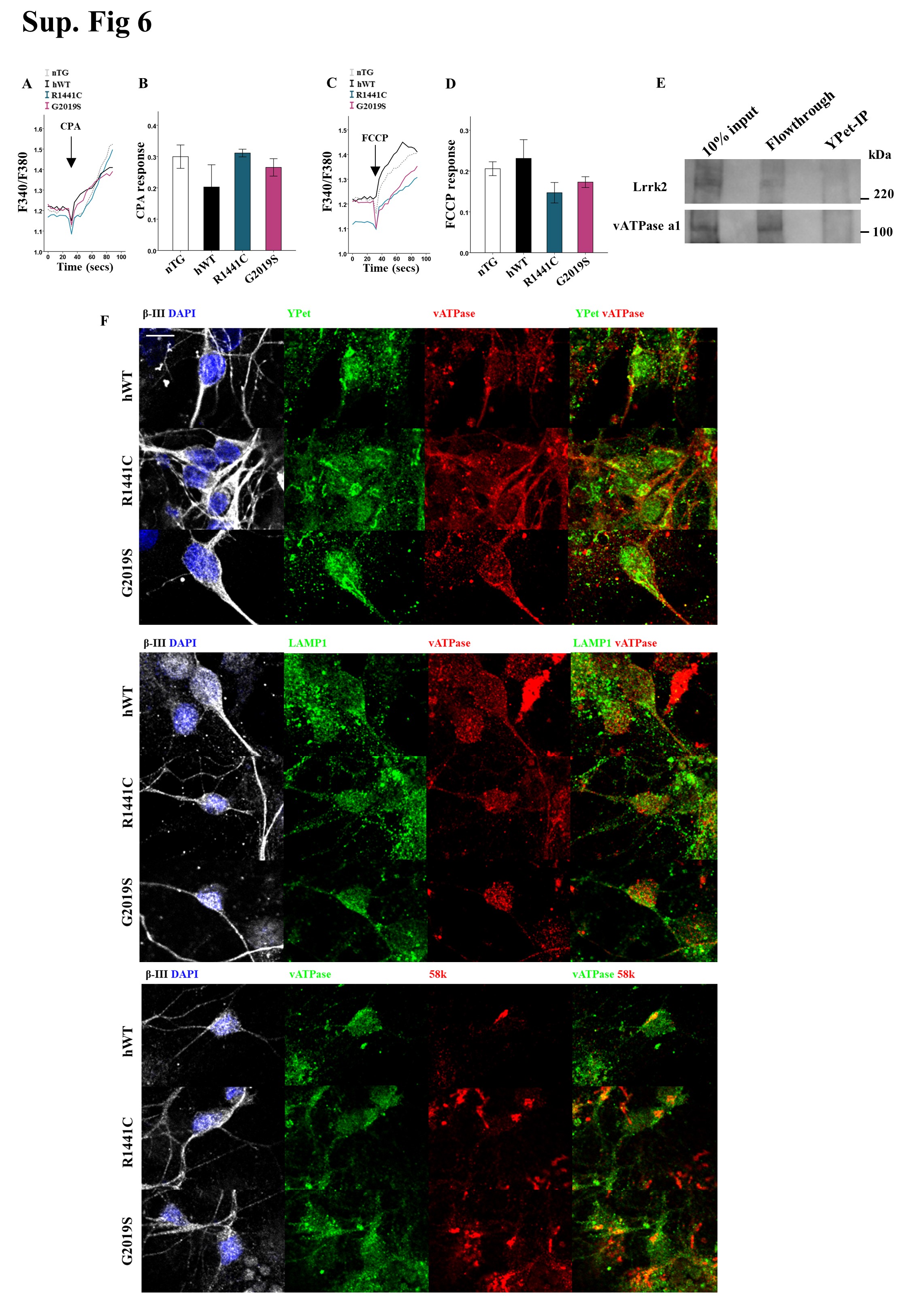

Supplement: Supp_ddz088 [file supp_ddz088.zip › Slide14.JPG]

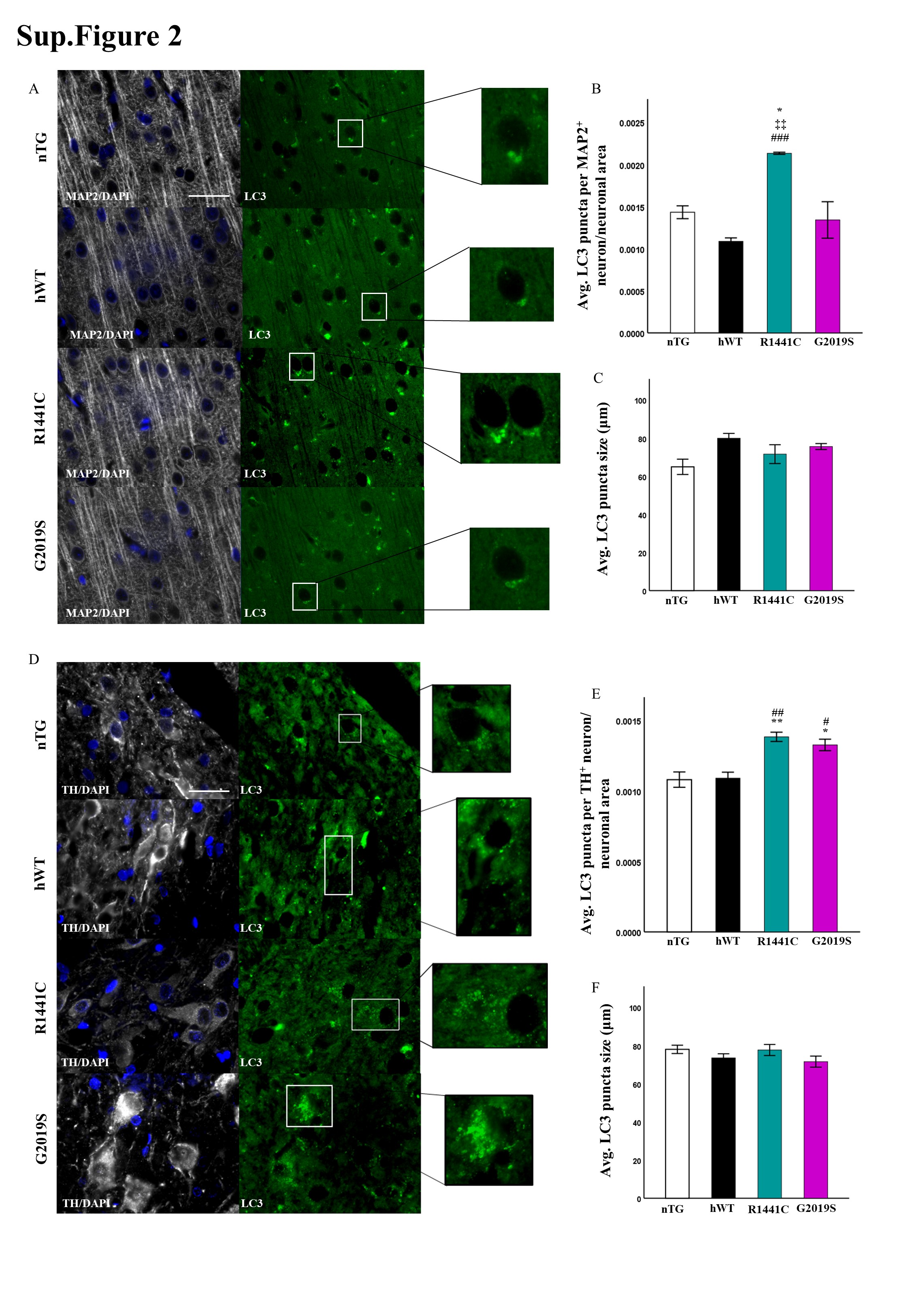

Supplement: Supp_ddz088 [file supp_ddz088.zip › Slide10.JPG]

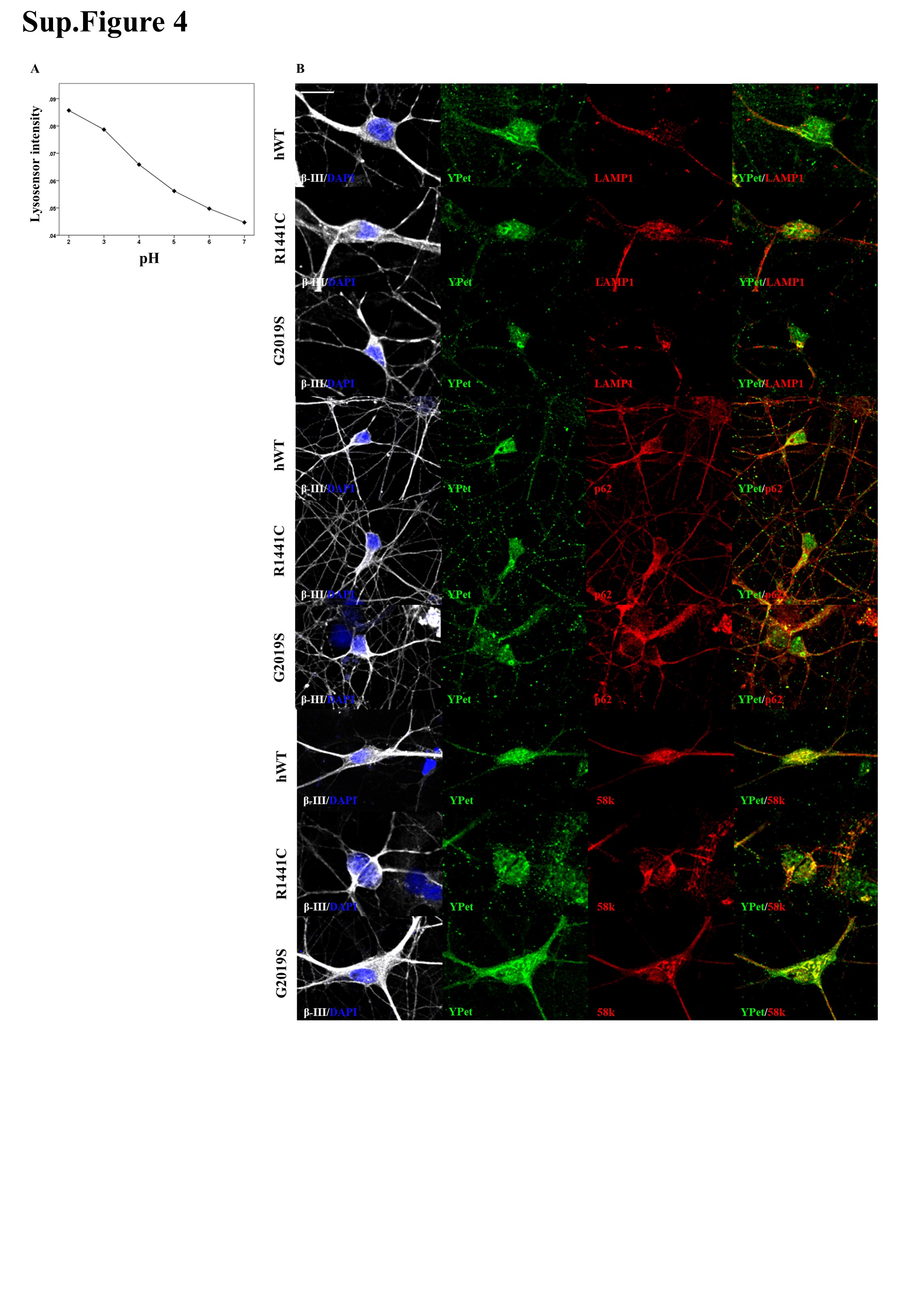

Supplement: Supp_ddz088 [file supp_ddz088.zip › Slide12.JPG]

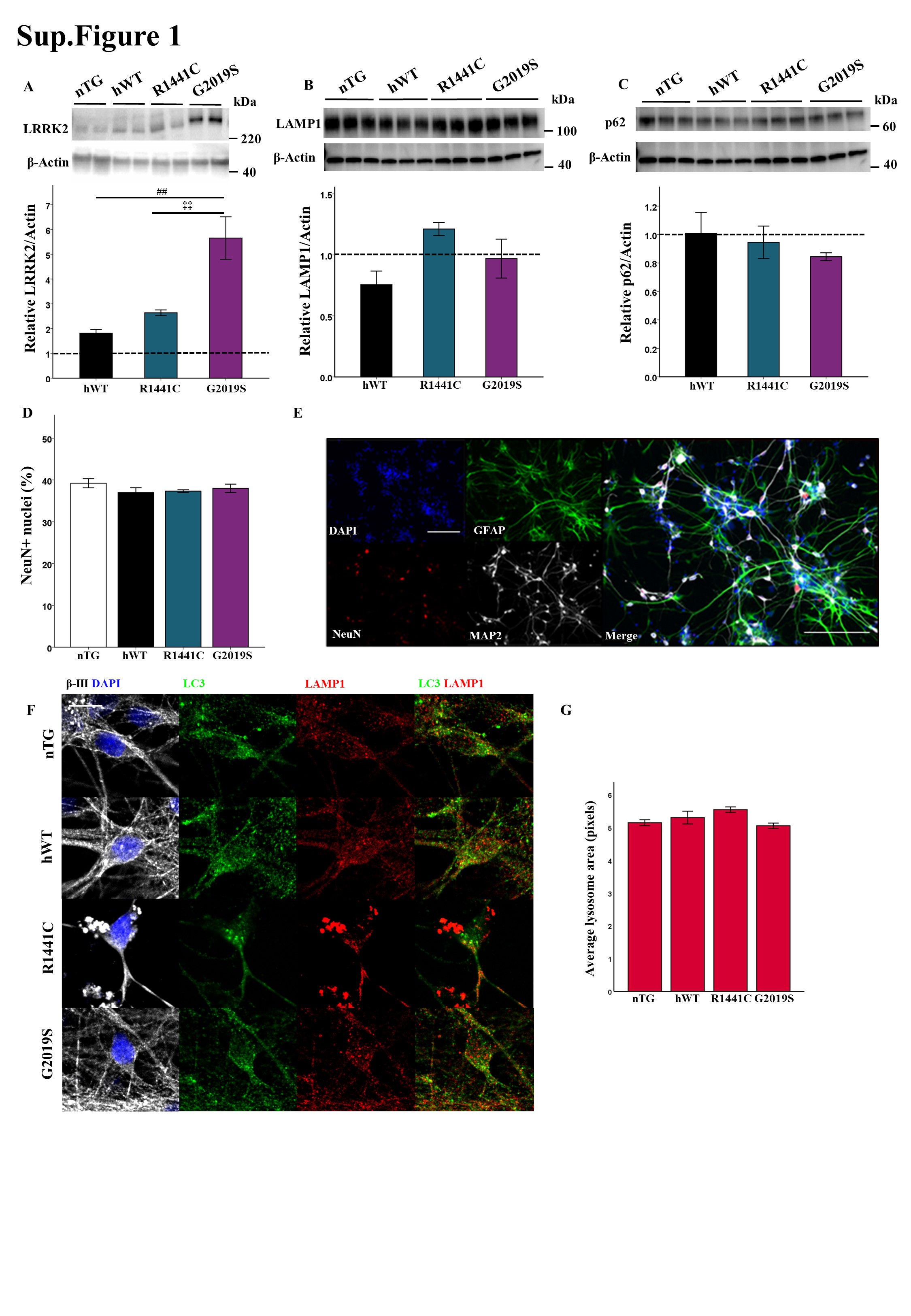

Supplement: Supp_ddz088 [file supp_ddz088.zip › Slide9.JPG]

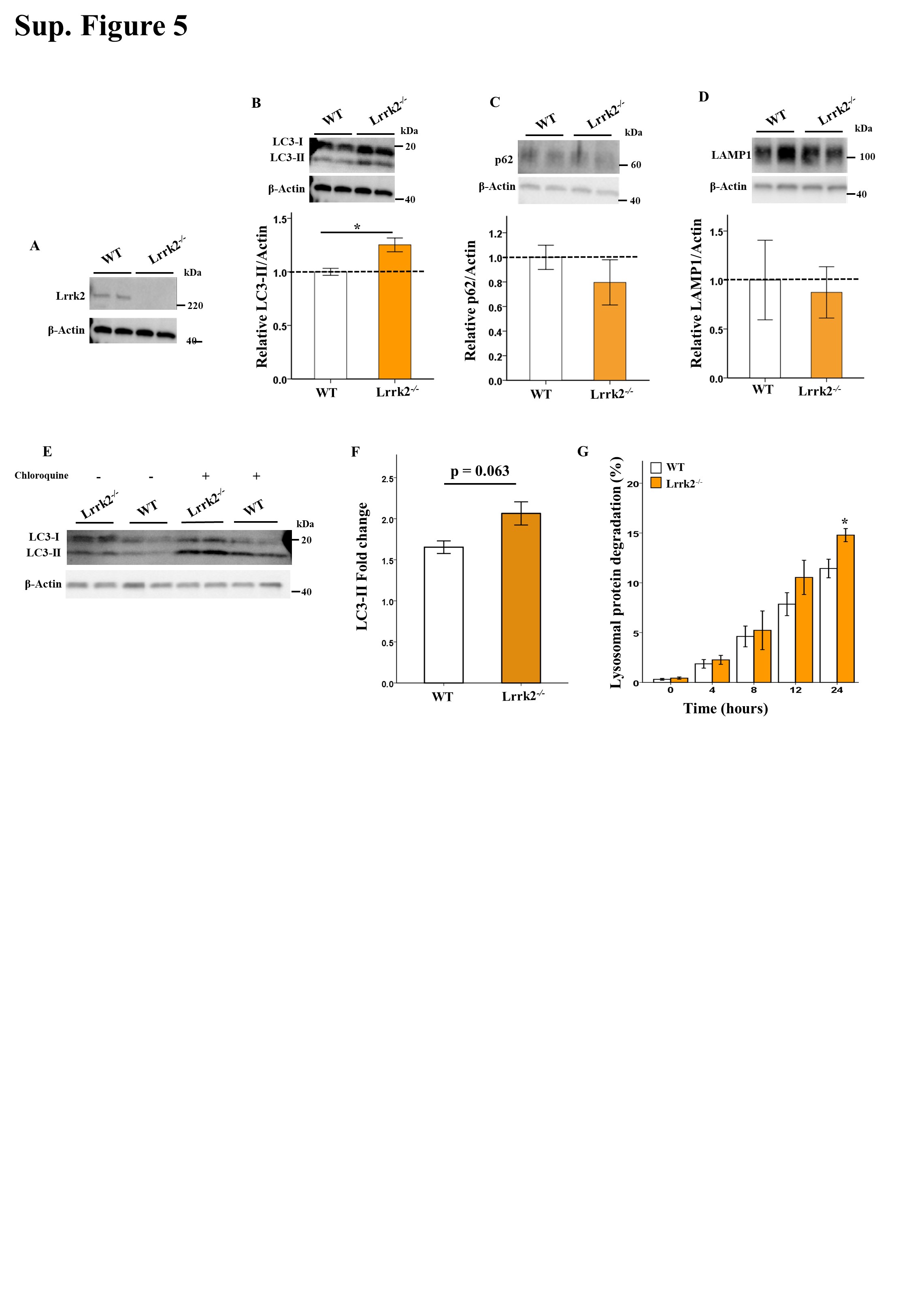

Supplement: Supp_ddz088 [file supp_ddz088.zip › Slide13.JPG]

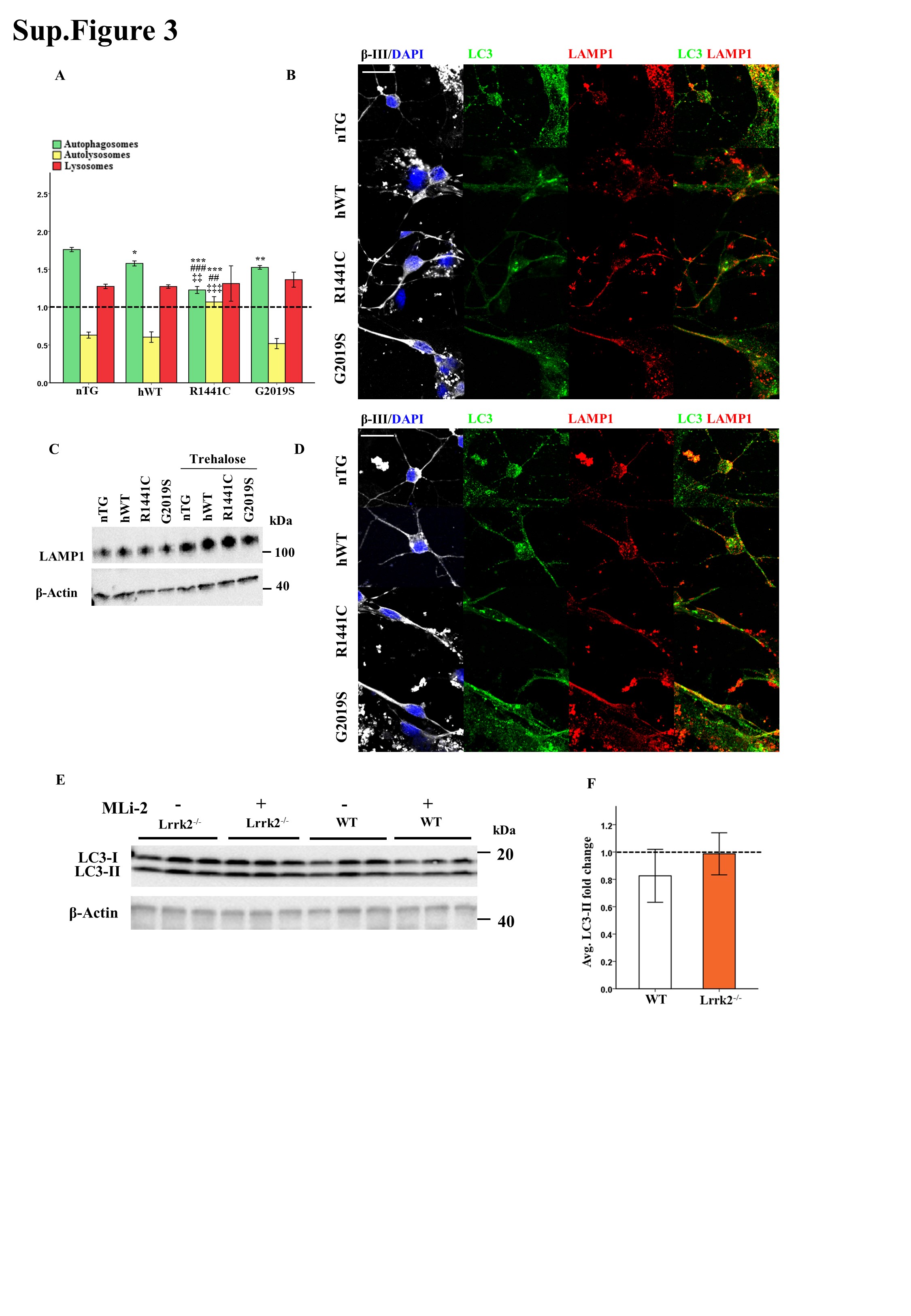

Supplement: Supp_ddz088 [file supp_ddz088.zip › Slide11.JPG]
